# Inhibition Effect and Mechanism of Phenolic Antioxidants on Coal Spontaneous Combustion

**DOI:** 10.1021/acsomega.5c11231

**Published:** 2026-01-16

**Authors:** Yunfei Liu, Yan Shang, Yu Jing, Jiafei Zhang, Yongfeng Jia

**Affiliations:** † School of Civil Engineering, 384127 Ordos Institute of Technology, Ordos,017000, China; ‡ Ordos Mining Area Geohazard Prevention and Geoenvironmental Protection Engineering Research Center, Ordos, 017000, China

## Abstract

Coal spontaneous combustion is a major safety and environmental concern in mining operations. This study systematically evaluated three phenolic antioxidants (BHA, BHT, and PG) as promising inhibitors through multiscale experiments and quantum chemical calculations. Results showed that all antioxidants delayed the coal–oxygen reaction by increasing characteristic temperatures and reducing heat release. For instance, the initial exothermic temperature T_2_ was increased by 21.7 °C, 13.71 °C, and 5.02 °C for PG, BHT, and BHA, respectively. A novel comprehensive evaluation system, based on the coal spontaneous combustion risk coefficient (C_r_) and destructive coefficient (C_d_), identified PG as the most effective inhibitor overall. Analyses via SEM and LTNA showed that PG treatment reduced the coal’s specific surface area and pore volume, leading to a densification of its microstructure. This physical alteration contributes an additional oxygen-blocking effect. EPR and in situ FTIR confirmed that these antioxidants act as hydrogen donors, effectively quenching active free radicals (e.g., ·OH, CH_3_·) and suppressing the conversion of carbon-centered to oxygen-centered radicals, thereby terminating chain reactions. Quantum chemical calculations corroborated that the reactions between the antioxidants and key radicals are spontaneous and exothermic. The reactivity order was determined as ·OH > CH_3_· > Ar–CH_2_· > Ar–CH_2_–O· > Ar–CH_2_–OO. These results elucidated the underlying structure–activity relationship: PG’s superior performance is directly attributable to its three phenolic hydroxyl groups, which provide a greater total radical scavenging capacity compared to the single group in BHA or BHT. This study confirms that PG is an efficient, low-cost, and environmentally friendly inhibitor with strong potential to prevent coal spontaneous combustion, providing a theoretical basis for its industrial application.

## Graphical abstract

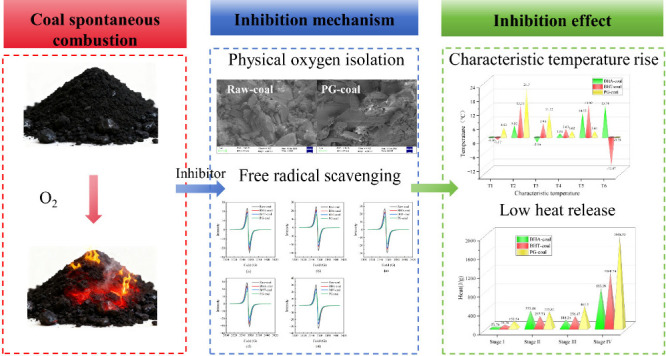

## 1 Introduction

Given China’s resource endowment characterized by abundant coal, scarce oil, and limited gas, coal is projected to maintain a 50% share in the primary energy mix by 2050.^1^ This indicates that coal will continue to serve as the ballast and stabilizer of China’s energy security system for the foreseeable future.^2^ However, spontaneous combustion accidents occur frequently during coal mining, posing threats to workers’ lives, damaging the regional ecological environment, and thereby severely restricting safe and efficient mining.^3^ The essence of spontaneous coal combustion lies in a chain reaction of coal-oxygen compounds.^4^ Macroscopically, this process manifests as the heat release rate exceeding the heat dissipation rate during oxidation.^5^ Microscopically, it is initiated by free radicals generated from the oxidation of active functional groups, which then propagate the chain reaction. Inhibitors represent one of the key technologies for preventing spontaneous coal combustion, with their core function being to delay or interrupt the coal-oxygen reaction.^6^ Based on their inhibition mechanisms, commonly used inhibitors can be divided into two categories: physical inhibitors and chemical inhibitors.

Physical inhibitors, such as halide salts,^7^ Phosphorus-based compounds,^8^ and hydroxides,^9^ primarily function by isolating oxygen or through endothermic cooling. However, limited by environmental conditions and their intrinsic material properties, these inhibitors often suffer from short effective durations and low efficiency. They are typically only effective in the initial stages of the coal-oxygen reaction, making them inadequate for the long-term and deep-seated fire prevention and control demands of coal mines. For instance, an aqueous solution of MgCl_2_ inhibits combustion effectively at low temperatures (usually below 180 °C) by isolating oxygen and absorbing heat through water evaporation.^10,11^ Nevertheless, it rapidly loses efficacy at elevated temperatures due to dehydration.^12^

To further enhance inhibition efficiency, scholars have proposed chemical inhibitors targeting the free radical chain reaction mechanism.^4,13,14^ Common chemical inhibitors include organic salts and antioxidants. Gao et al.^15^ studied the inhibitory effect of sulfamic acid on coals of different metamorphic grades. Their results indicated that sulfamic acid exhibited a strong inhibitory effect on middle and low rank coals. It functions through two primary mechanisms: (1) acid corrosion, which damages inorganic minerals and organic macromolecules in coal, thereby altering its pore structure; (2) hydrolysis, which generates noncombustible gases to inactivate free radicals and hinder the coal-oxygen reaction. To overcome the issues of weak efficacy and short duration associated with single-component inhibitors, Pan et al.^16^ proposed a novel composite inhibitor consisting of luteolin, tea polyphenols, and urea (LTU). They compared the inhibition effectiveness and mechanisms of LTU with those of traditional inhibitors (NaHCO_3_ and VC). The results demonstrate that the components within LTU exhibit a significant synergistic effect. The physical components of LTU isolate oxygen and absorb heat to lower temperature, while its chemical components consume active groups (such as −OH, RO·, and ROO·) to inhibit oxidation and block chain reactions. LTU can significantly reduce the generation of CO and CH_4_ and increase the production temperatures of these gases. Under identical conditions, the inhibitory performance of LTU surpasses that of both NaHCO_3_ and VC. Gao et al.^17^ investigated the inhibitory effect and mechanism of glutathione on coal spontaneous combustion by combining experimental research with quantum chemical calculation. The results show that glutathione, acting as a hydrogen donor, can neutralize carbon-centered and peroxy free radicals. This reduces the concentration and reactivity of free radicals throughout the chain reaction process, thereby blocking the coal-oxygen reaction.

Chemical inhibitors have attracted extensive attention from researchers due to their superior performance. However, their practical application faces several limitations. For example, the action of sulfamic acid depends on acid corrosion and hydrolysis, which may damage equipment and pollute the mine water environment. The inhibitory effect of urea weakens above 200 °C and may even catalyze oxidation. Natural antioxidants, such as tea polyphenols, luteolin, and glutathione, are expensive, making them difficult to apply on a large industrial scale. Therefore, the development of efficient, environmentally friendly, and low-cost inhibitors has become a key breakthrough direction for preventing coal spontaneous combustion.

Based on the foregoing discussion, three phenolic antioxidants—propyl gallate (PG), butylhydroxyanisole (BHA), and butylated hydroxytoluene (BHT)—were selected as novel inhibitors for systematic study in preventing coal spontaneous combustion. These compounds are widely used in the food industry and have a mature commercial foundation.^18–21^ Their advantages include: (1) Cost-effectiveness and easy availability: As bulk industrial products, their cost is significantly lower than that of most natural antioxidants. (2) Safety and environmental friendliness: Being food additives, they have comprehensive toxicological data and pose low environmental risks. (3) Effective radical quenching mechanism: Their phenolic hydroxyl structures can efficiently quench peroxy radicals (ROO·) by donating hydrogen atoms, which aligns perfectly with the requirement to interrupt the chain reaction in coal spontaneous combustion. Furthermore, the differences in their molecular structures—such as the steric hindrance in BHT, the electron-donating group in BHA, and the polyhydroxyl structure of PG—provide an ideal model for further investigating the structure–activity relationship of phenolic antioxidants.^22,23^

This study employed a multiscale experimental approach combined with quantum chemical calculations. The macro-inhibitory effect was evaluated through differential scanning calorimetry (DSC) and temperature-programmed oxidation (TPO). The microstructure was characterized using scanning electron microscopy (SEM) and low-temperature nitrogen adsorption (LTNA). Electron paramagnetic resonance (EPR) and in situ Fourier transform infrared spectroscopy (in situ FTIR) were utilized to monitor the concentration of free radicals and the dynamic evolution of key active functional groups. Furthermore, quantum chemical calculations were conducted to elucidate the inhibition mechanisms of PG, BHA, and BHT at the molecular level. This integrated methodology is designed to systematically clarify the inhibition mechanisms of these phenolic antioxidants on coal spontaneous combustion, thereby providing a theoretical foundation for their future industrial application in coal fire prevention and control.

## 2 Materials and Methods

### 2.1 Sample Preparation

The coal samples used in this study were a long-flame coal collected from the Selian No. 2 mine (Dongsheng District, Ordos City, Inner Mongolia). Upon collection, the samples were immediately sealed, transported to the laboratory, and ground to pass through a 100-mesh sieve (with an aperture of 150 um) for subsequent experiments. Proximate analyses of the coal were performed, and the results were summarized in Table 1. BHA, BHT, and ethanol (all of Analytical Reagent grade, >98% purity) were purchased from McLean Biochemical Technology Co., Ltd. (Shanghai, China). PG (Analytical Reagent grade, > 98% purity) was purchased from Ron Chemical Reagent Co., Ltd. (Shanghai, China).

**Table 1:** Proximate Analyses of the Coal

**M**_**ad**_	**A**_**ad**_	**V**_**ad**_	**FC**_**ad**_
3.8%	7.1%	31.8%	57.3%

Ethanol was utilized as a solvent to prepare solutions of BHA, BHT, and PG due to their low solubility in water. Each inhibitor solution was prepared by dissolving 6 g of the antioxidant (BHA, BHT, or PG) in 44 g of absolute ethanol, yielding a 12 wt % solution. For the inhibition treatment, 50 g coal samples were thoroughly mixed with the respective antioxidant solutions at a solid-to-solution ratio of 1:1 (g:g). After a 48 h inhibition period, the samples were dried at 38 °C in a drying oven until a constant mass was achieved, ensuring the removal of excess moisture. The resulting coal samples were labeled as BHA-coal, BHT-coal, and PG-coal. As a control, raw coal samples underwent the same treatment procedure and were labeled as Raw-coal. The preparation of the sample and the corresponding experimental procedure are illustrated in Figure 1.

**Figure 1:**
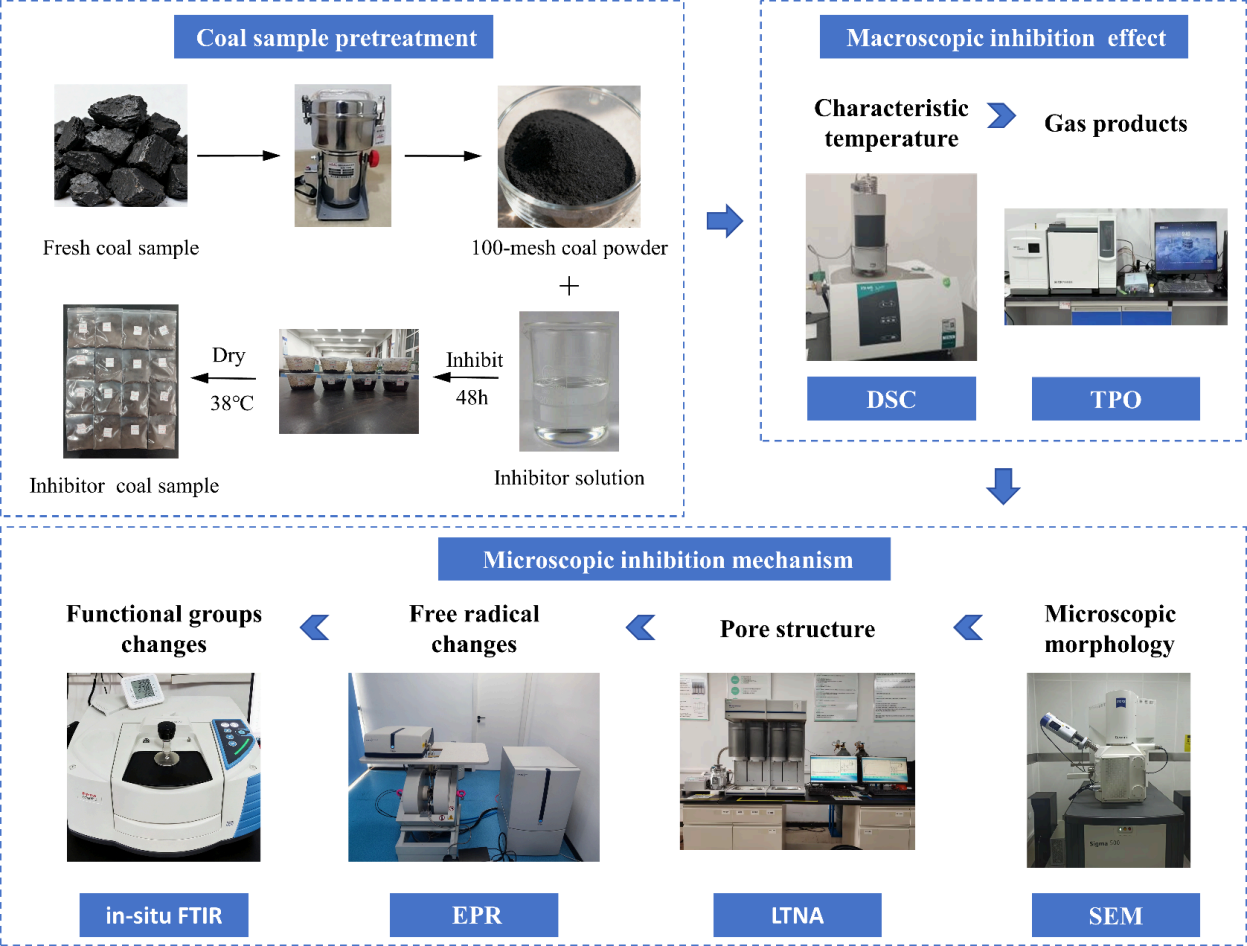
Schematic diagram of experimental equipment and procedures.

### 2.2 Differential Scanning Calorimetry (DSC)

DSC was conducted on a Netzsch STA 449 F3 simultaneous thermal analyzer. Approximately 10 mg of each sample was heated from 30 to 800 °C at a constant heating rate of 10 °C/min under a dry air atmosphere with a flow rate of 100 mL/min.

### 2.3 Temperature-Programmed Oxidation (TPO)

The temperature-programmed oxidation system mainly consisted of an air inlet system, a ZRD-II temperature-programmed furnace, and a GC-4000A gas chromatography workstation, which was used to monitor the oxidation products during the low-temperature oxidation of coal. The experimental conditions were set as follows: the gas atmosphere was air with a flow rate of 100 mL/min. The temperature program for the furnace was as follows: ramping from room temperature to 30 °C in 10 min, holding at 30 °C for 20 min, and then heating to 220 °C at a constant rate of 1 °C/min. Gas samples were collected at intervals: every 10 °C from 30 to 100 °C, and every 20 °C from 100 to 220 °C.

### 2.4 Scanning Electron Microscopy (SEM)

SEM was performed on a Zeiss Sigma 500 instrument to analyze the surface morphology of the raw and inhibited coal samples. Before analysis, all samples were sputter-coated with a gold layer to improve conductivity.

### 2.5 Low-Temperature Nitrogen Adsorption (LTNA)

The pore structure of the coal samples was analyzed by low-temperature nitrogen adsorption using a Micromeritics ASAP 2460 analyzer. Before the measurements, the samples were degassed at 105 °C for 6 h to remove physically adsorbed water and impurities. Nitrogen adsorption isotherms were then measured at 77 K across a relative pressure (P/P_0_) range of 0.01 to 0.99.

### 2.6 Electron Paramagnetic Resonance Spectroscopy (EPR)

EPR was employed to determine the free radical concentrations in the raw coal and inhibited coal samples at various temperatures (40, 80, 120, 160, and 200 °C) to assess their inhibitory effects. Measurements were conducted on a Bruker EMXplus-6/1 under the following conditions: center field, 3360 G; sweep width, 100 G; time constant, 0.01 ms; sweep time, 30 s; microwave power, 3.17 mW; microwave frequency, 9.41 GHz; modulation amplitude, 1 G; and modulation frequency, 100 kHz.

### 2.7 In Situ Fourier Transform Infrared Spectroscopy (in Situ FTIR)

The evolution of functional groups in the raw and inhibited coal samples during low temperature oxidation was monitored using in situ FTIR on a Bruker INVENIO S spectrometer. Spectra were acquired in Kubelka–Munk format over a spectral range of 600–4000 cm^–1^ with a resolution of 4 cm^–1^ and 32 scans per spectrum. The experiment was conducted under a flowing dry air atmosphere (50 mL/min) while the temperature was raised from 30 to 200 °C at a rate of 5 °C/min. Data collection was triggered at predefined temperatures (40, 80, 120, 160, and 200 °C), with a 5 min isothermal hold at each temperature prior to measurement.

### 2.8 Quantum Chemical Calculation

To elucidate the inhibition mechanism of phenolic antioxidants, molecular models of the antioxidants and key active radicals were constructed using GaussView 6.0. These models were subsequently optimized, and their vibrational frequencies were calculated using Gaussian 16 at the B3LYP/6-311G­(d,p) level of theory. For the optimized structures, frontier orbital and thermodynamic analyses were then performed.

## 3 Results and Discussion

### 3.1 Thermal Analysis

#### 3.1.1 Characteristic Temperature

To verify the inhibitory effect of phenolic antioxidants on coal spontaneous combustion and compare their performance, DSC were carried out on raw coal and the inhibited samples (BHA-coal, BHT-coal, and PG-coal). The results, depicted in Figure 2, show that the DSC curves of all samples share a similar profile. As the temperature rises, the curves undergo a brief initial increase, followed by a gradual decline and a subsequent plateau. They then fall rapidly to the point of maximum heat release rate before eventually stabilizing.

**Figure 2:**
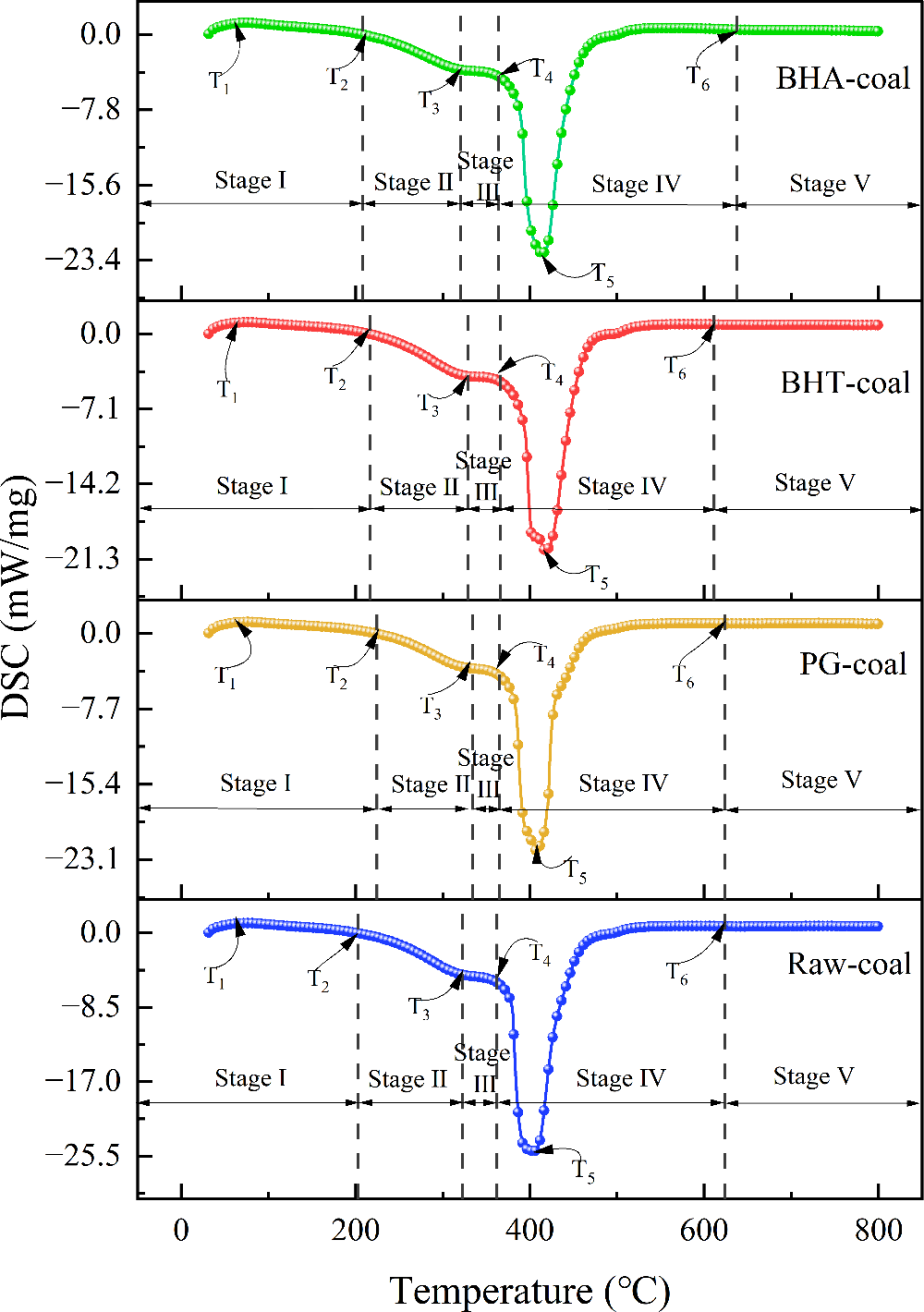
DSC curves of different coal samples.

Based on the DSC curves, the following characteristic temperatures were defined. During the initial phase of the coal-oxygen reaction, coal samples typically exhibit an endothermic effect due to water evaporation and gas desorption. The temperature at which the peak endothermic rate occurs is denoted as T_1_. As temperature rises, the exothermic rate of the coal samples gradually increases. T_2_ is defined as the temperature at which the exothermic rate matches the endothermic rate. Beyond T_2_, the exothermic rate exceeds the endothermic rate, and the coal-oxygen reaction exhibits an exothermic effect. T_3_ marks the onset of coal thermal decomposition, characterized by heat absorption that causes the exothermic curve to decelerate and plateau. T_4_ is the ignition temperature, where the fixed carbon and volatile components begin to combust. T_5_ represents the peak exothermic rate during combustion, and T_6_ is the burnout temperature, where the exothermic rate returns to zero. The characteristic temperatures for each coal sample are provided in Table 2. Based on these temperatures, the coal-oxygen reaction process is divided into five stages: Stage I (T_0_–T_2_): water evaporation and gas desorption; Stage II (T_2_–T_3_): chemical adsorption and slow oxidation; Stage III (T_3_–T_4_): coal pyrolysis; Stage IV (T_4_–T_6_): combustion of fixed carbon and volatile matter; Stage V (>T_6_): burnout.

**Table 2:** Characteristic Temperatures of Different Coal Samples

	**Characteristic temperatures (°C)**					
**Coal sample**	**T**_**1**_	**T**_**2**_	**T**_**3**_	**T**_**4**_	**T**_**5**_	**T**_**6**_
BHA-coal	68.59	207.79	320.69	363.79	415.09	637.59
BHT-coal	67.78	216.48	328.78	365.78	418.48	611.38
PG-coal	73.47	224.47	333.97	365.17	407.57	623.57
Raw-coal	68.95	202.77	322.85	362.15	404.56	623.85

As shown in Figure 3, the characteristic temperatures of the inhibited coal samples generally shift toward higher temperatures compared to raw coal, with the most significant increases observed for T_2_, T_3_, and T_5_. Specifically, the initial exothermic temperature T_2_ increases by 5.02 °C, 13.71 °C, and 21.7 °C for BHA-coal, BHT-coal, and PG-coal, respectively. For the decomposition temperature T_3_, PG-coal and BHT-coal increase by 5.93 and 11.12 °C, respectively, whereas BHA-coal decreases by 2.16 °C, indicating its lack of inhibition at this stage. Regarding the peak exothermic temperature T_5_, BHA-coal and BHT-coal show substantial increases of 10.53 and 13.92 °C, respectively, while PG-coal increases slightly by 3.01 °C.

**Figure 3:**
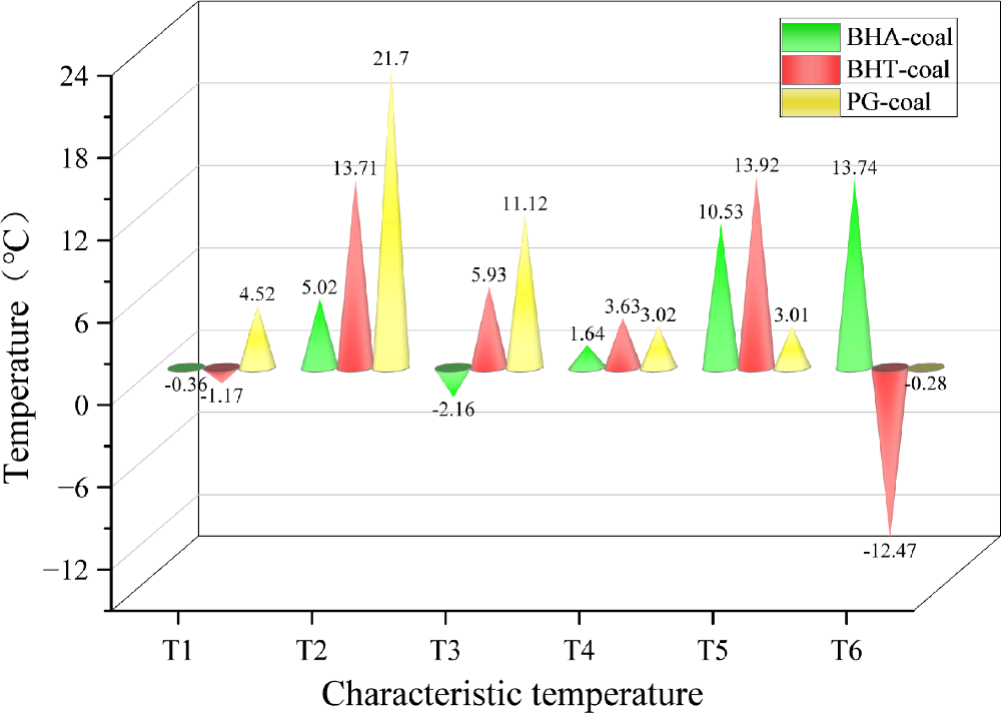
Increase in characteristic temperature of the inhibited coal samples relative to raw coal.

In summary, compared to raw coal, the inhibited coal samples exhibited delayed onset temperatures for key reactions such as oxidative exotherm and combustion, indicating a retardation of the coal-oxygen reaction process. PG significantly increased the characteristic temperatures T_2_ and T_3_, with a more modest effect on T_1_; this demonstrates its strong inhibitory effect in the early stages of the coal-oxygen reaction (prior to 320 °C). BHT effectively elevated T_2_, T_3_, and T_5_, and its effective temperature range was broader than that of PG. In contrast, BHA primarily increased T_5_ and T_6_, indicating that its main inhibitory function occurs during the combustion stage.

#### 3.1.2 Thermal Effect of Coal Oxidation

Heat release is the primary driver of coal spontaneous combustion. The fundamental mechanism involves thermal runaway caused by the heat released from the oxidation of active groups in the coal. When the accumulated heat raises the coal temperature above the ignition point, spontaneous combustion occurs. Furthermore, the heat release rate determines the resultant fire’s hazard severity; a higher rate leads to more intense combustion and thus greater safety and environmental impacts. To evaluate the inhibition effect of phenolic antioxidants on coal spontaneous combustion, the heat release at various stages was quantified. This was done by integrating the respective segments of the DSC curve (Figure 2) between the characteristic temperatures, with the baseline calibrated to zero. The calculated results are summarized in Table 3.

**Table 3:** Heat Release at Different Stages of Coal Oxygen Composite Reaction

	**Heat release** **(J/g)**			
**Coal sample**	Stage I	**Stage II**	**Stage III**	**Stage IV**
BHA-coal	–719.8	1160.22	1002.65	6767.89
BHT-coal	–760.8	1277.57	926.44	6452.54
PG-coal	–838.58	1179.49	721.61	5672.75
Raw-coal	–686.04	1535.3	1182.91	7621.28

Figure 4 presents the reduction in heat release of the inhibited coal samples compared to the raw coal across different reaction stages. In Stage I, all three inhibited samples exhibited increased heat absorption, with PG-coal showing the most pronounced effect at 152.54 J/g above raw coal. During Stages II and III, the heat release from each inhibited sample was 200–400 J/g lower than that of raw coal, indicating that the inhibitors effectively mitigate heat accumulation prior to combustion, thereby delaying the coal oxygen composite reaction process and reducing spontaneous combustion risk. In Stage IV, heat release was substantially suppressed. Specifically, reductions of 853.39 J/g, 1168.74 J/g, and 1948.83 J/g were observed for BHA-coal, BHT-coal, and PG-coal, respectively. These results unequivocally demonstrate the efficacy of phenolic antioxidants in lowering combustion intensity and mitigating the associated hazards of coal spontaneous combustion.

**Figure 4:**
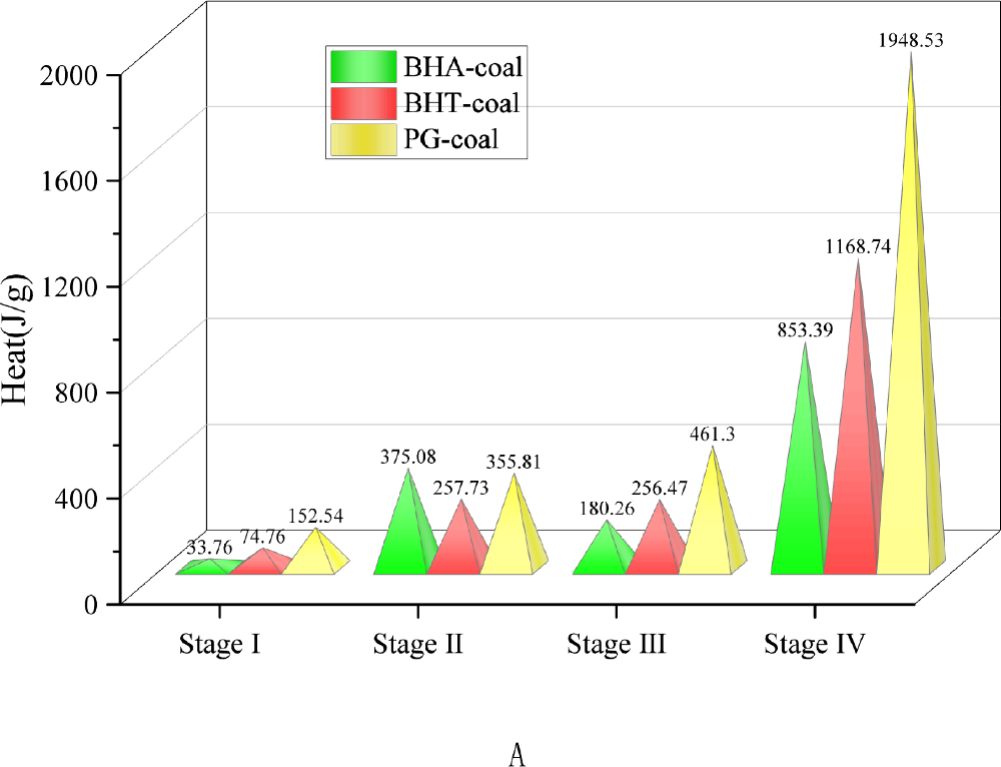
Reduction in heat release of the inhibited coal sample compared with raw coal across different reaction stages.

#### 3.1.3 Comprehensive Evaluation of Inhibition Effect

The analysis in Sections 3.1.1 and 3.1.2 reveals the limitations of relying on a single metric, such as characteristic temperature or reaction heat, to evaluate inhibitor efficacy. For instance, PG-coal exemplifies this limitation. From the perspective of characteristic temperature, PG exhibits no significant improvement beyond the ignition point, indicating that its inhibition is confined to the early stages of the coal-oxygen complex reaction. However, from the perspective of heat release, PG substantially reduces the heat release during the combustion stage, demonstrating its effective inhibition in this phase.

To address the limitations of single-parameter evaluations, this study introduces two novel indices: the coal spontaneous combustion risk coefficient (C_r_) and the coal spontaneous combustion destructive coefficient (C_d_). The primary significance and advantages of these indices lie in their integrated and targeted design. C_r_ quantifies the likelihood of ignition, increasing with either a shorter time to reach the ignition temperature or greater heat release during the oxidation stage. C_d_ assesses the severity of an established fire, which escalates with greater total heat release and a faster release rate during combustion. Based on these principles, C_r_ and C_d_ can be calculated using equations 1 and 2,^24,25^ respectively.

$$C_{r}=10^{5}\frac{Q_{ig}}{\left(t_{2}-t_{1}\right)T_{2}T_{4}}$$ 1

$$C_{d}=q_{\max}\frac{Q_{\max}}{\left(t_{5}-t_{4}\right)}$$ 2

The symbols in the equations are defined as follows: T_2_, T_4_, and T_5_ represent the initial exothermic temperature, ignition temperature, and the temperature at the peak heat release rate, with corresponding times t_2_, t_4_, and t_5_, respectively. The thermal parameters are Q_ig_, the total heat release prior to T_4_; *q*_max_, the peak exothermic rate; and *Q*_max_, the total heat release during the combustion phase.

Compared to conventional parameters, the core advantage of C_r_ and C_d_ is their ability to integrate multiple, sometimes contradictory, thermal parameters into two indices with clear physical meanings. This provides a more holistic and practical framework for inhibitor evaluation, moving beyond isolated observations to comprehensive assessment.

As shown in Figure 5, C_r_ and C_d_ of the inhibited coal samples are lower than those of raw coal. This demonstrates that phenolic antioxidants can reduce both the risk of spontaneous combustion initiation and the severity of the resultant hazard. Crucially, the C_r_-C_d_ evaluation system reveals differential inhibitor performance that single metrics might obscure: Regarding the reduction of spontaneous combustion risk, the inhibitory efficacy of the three antioxidants ranks as PG > BHA > BHT; In terms of mitigating the destructive severity, the ranking is PG > BHT > BHA. Overall, PG exhibits the most comprehensive inhibitory effect among the three antioxidants.

**Figure 5:**
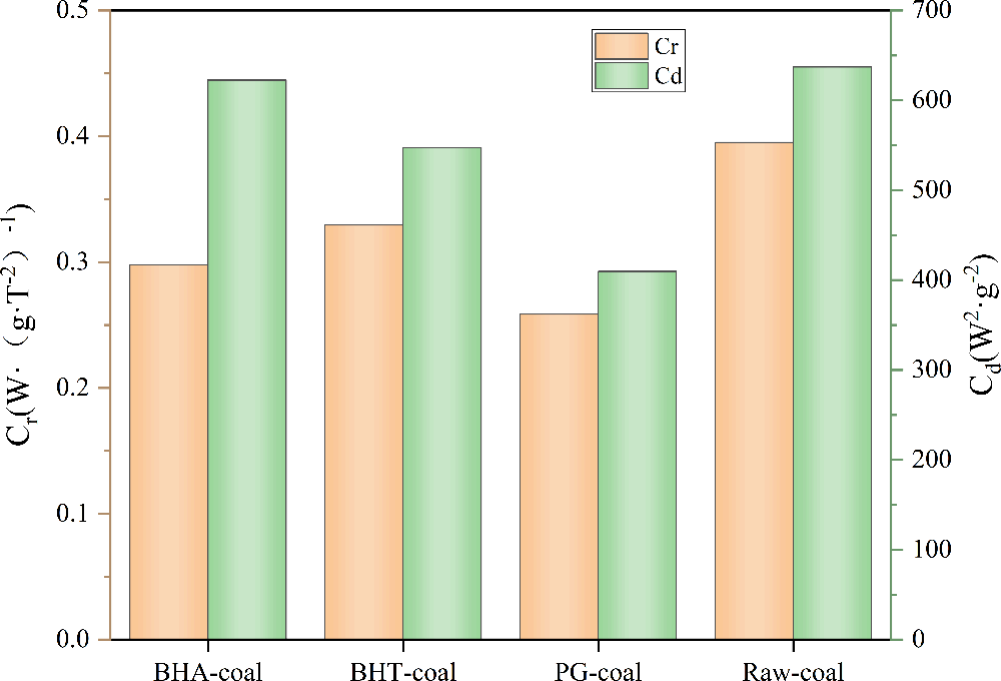
Risk coefficient and destructive coefficient of different coal samples.

### 3.2 Effect of Inhibitor on Oxidation Products

During coal oxidation, free radicals react with oxygen to produce a variety of gaseous products, such as CO, CO_2_, CH_4_. CO is commonly used as an indicator gas to characterize the intensity of the coal-oxygen reaction.^17,26^ As shown in Figure 6, the CO release from all coal samples increases with temperature, following a consistent overall trend. Below 100 °C, the amount of CO released is small, with each sample remaining under 1000 ppm. Above 100 °C, the oxidation reaction intensifies, leading to substantial CO generation and a rapid increase in its release rate with rising temperature. At any given temperature, the CO release from the three inhibited coal samples was lower than that from the raw coal. This indicates that the antioxidants effectively suppressed spontaneous combustion, with an inhibition efficacy order of PG > BHT > BHA. It is consistent with the results of thermal analysis.

**Figure 6:**
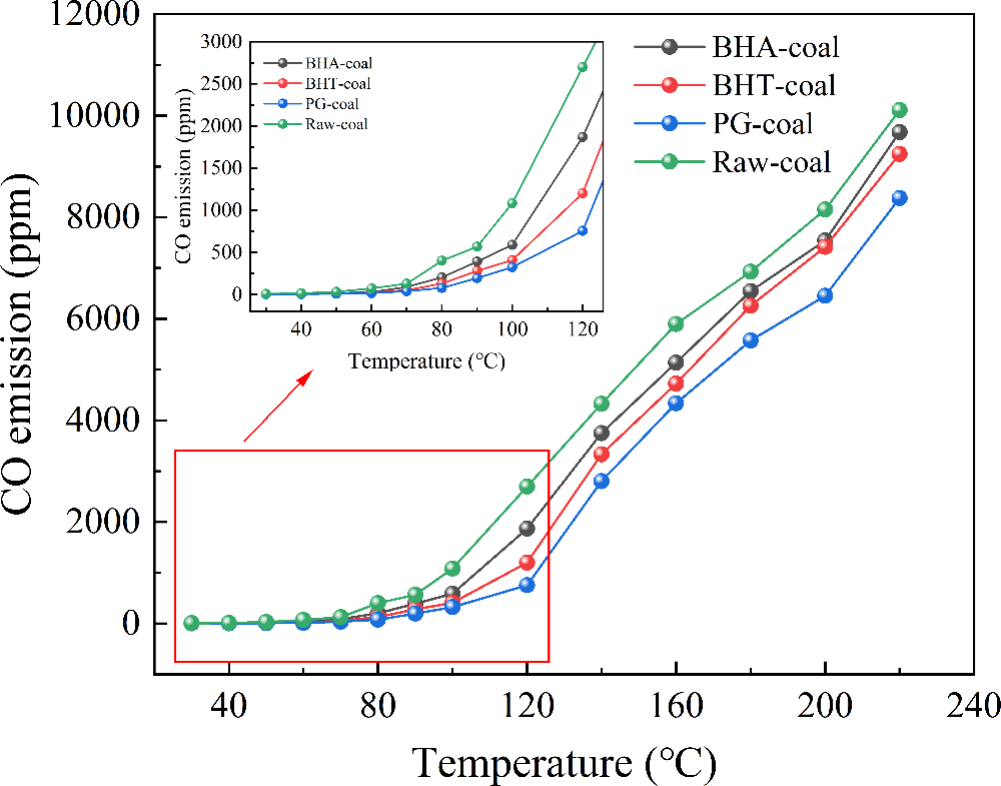
CO emission from different coal samples.

To quantitatively evaluate the inhibition effect of different antioxidants, the inhibition rate is used as the evaluation index, defined as the relative change in CO release from coal samples before and after inhibition.

$$E=\frac{A_{CO}-B_{CO}}{A_{CO}}$$ 3

where E is the inhibition rate (%), A_CO_ is the CO released from the raw coal (ppm), and B_CO_ is the CO released from the inhibited coal sample (ppm).

Figure 7 shows the variation in the inhibition rates of the three antioxidants with temperature. At reaction temperatures below 120 °C, the coal-oxygen reaction proceeds slowly, generating only a limited number of free radicals. Within this range, the antioxidants effectively capture active free radicals on the coal surface (such as ·OH and ·OOH), thereby delaying the formation of CO. The inhibition rates of BHA, BHT, and PG in this temperature range are 19.2–49.4%, 20–67.7%, and 49.8–80.8%, respectively.

**Figure 7:**
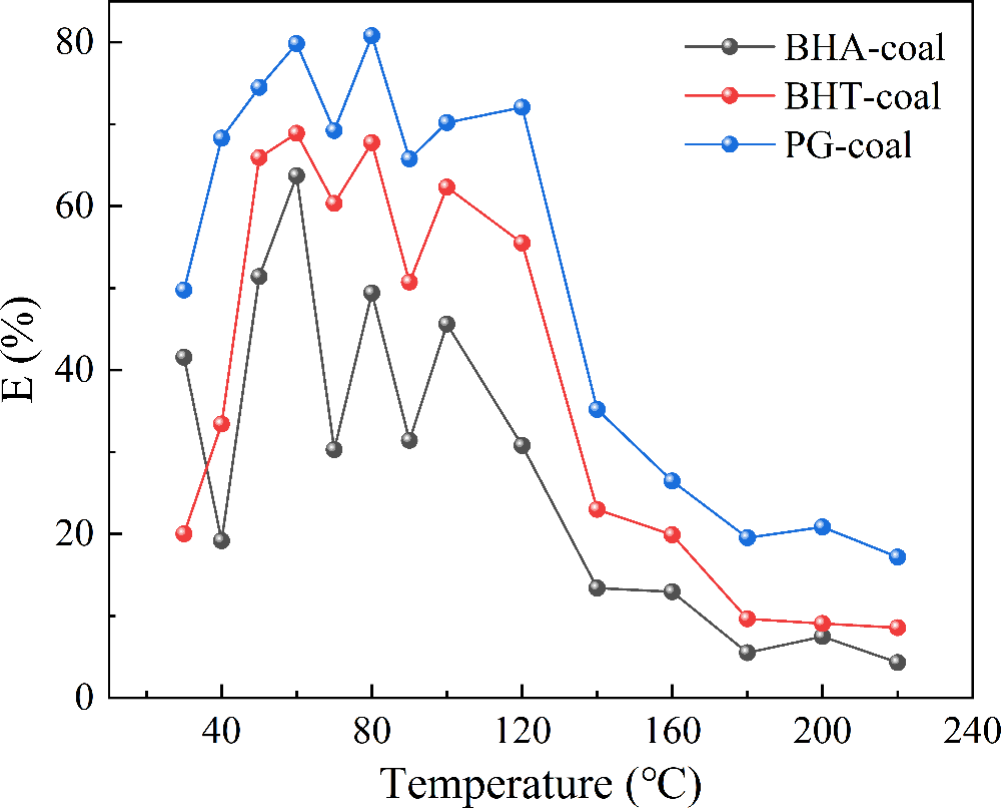
Inhibition rate of different antioxidants.

When the temperature exceeds 120 °C, the free radical chain reaction intensifies with increasing temperature. The accelerated consumption of antioxidants leads to a decline in their inhibitory efficacy. Beyond 180 °C, the inhibition rates of all three antioxidants gradually level off. At 220 °C, the inhibition rates of BHA, BHT, and PG are 4.3%, 8.6%, and 17.2%, respectively.

### 3.3 Micromorphology and Pore Characteristics

#### 3.3.1 Micromorphology

As shown in Figure 8, the raw coal microstructure exhibits distinct granularity, a high degree of fragmentation, disordered particle distribution, and a well-developed pore structure. The overall morphology of BHA-coal and BHT-coal is similar to that of raw coal. However, BHA-coal shows a significantly higher proportion of scale-like large particles, whereas BHT-coal contains a greater proportion of small particles with a more uniform size distribution. In contrast, the microstructure of PG-coal is markedly different, exhibiting a relatively smooth surface, an overall lamellar structure, and a poorly developed pore structure.

**Figure 8:**
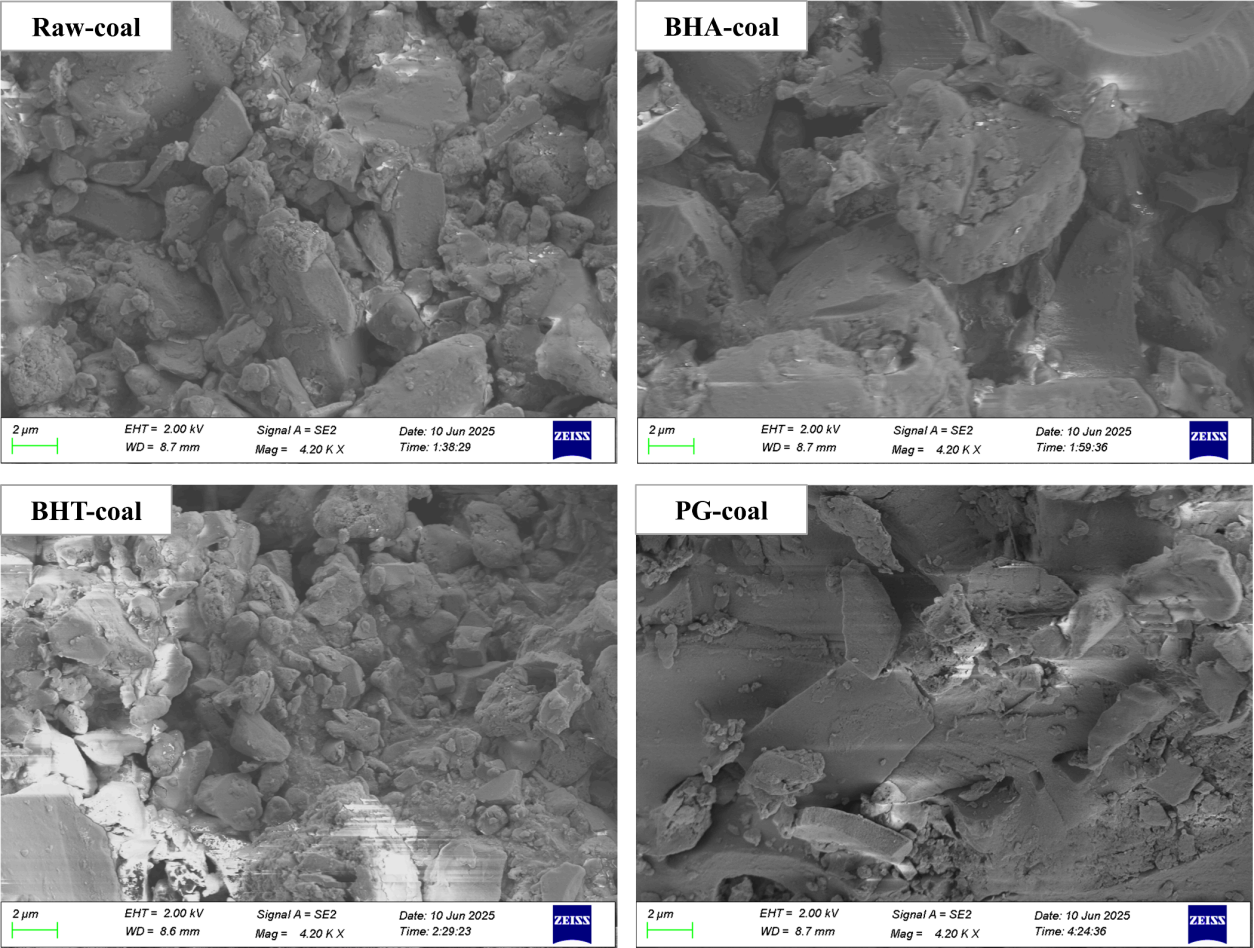
SEM images of different coal samples.

#### 3.3.2 Pore Characteristics

To further investigate the pore characteristics, the pore structure and specific surface area of the coal samples were analyzed using LTNA. As shown in Figure 9, the cumulative pore volumes of the three inhibited coal samples exhibited a similar trend to the raw coal as a function of pore width.

**Figure 9:**
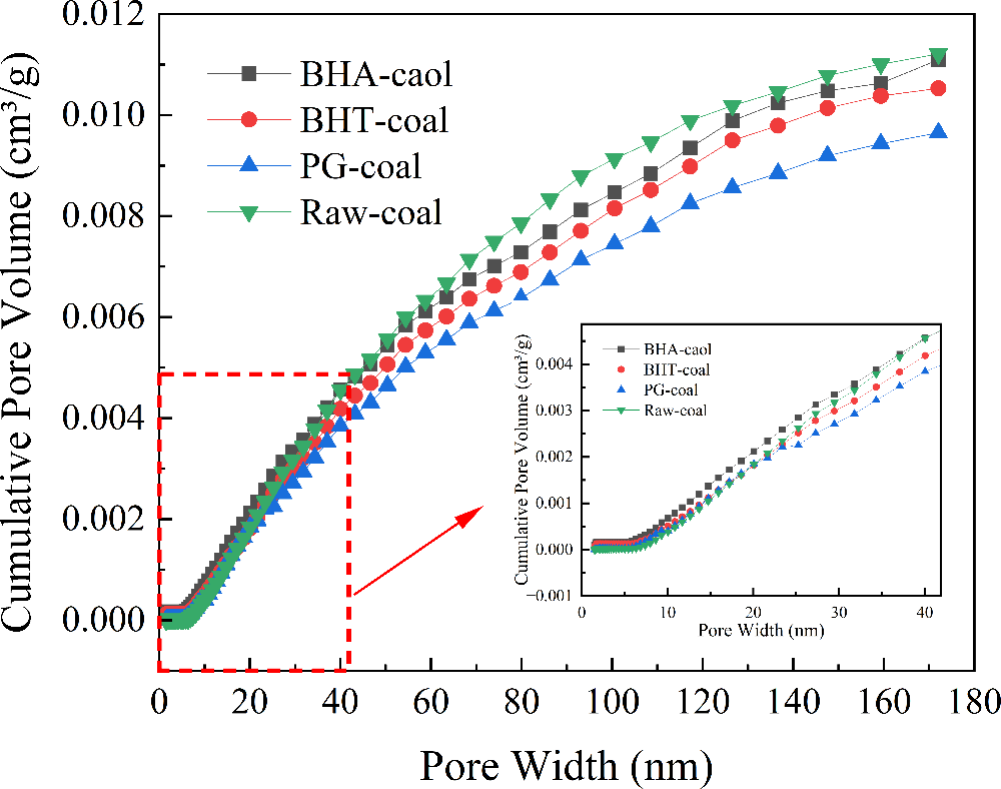
Cumulative pore volume of different coal samples.

According to Table 4, when the pore width was below 20 nm, the cumulative pore volumes of BHT-coal and PG-coal were 1.82 × 10^–3^ and 1.85 × 10^–3^cm^3^/g, respectively, which were similar to that of the raw coal (1.84 × 10^–3^ cm^3^/g). In contrast, the cumulative pore volume of BHA-coal was 2.12 × 10^–3^ cm^3^/g higher than that of the raw coal. When the pore size exceeded 20 nm, the cumulative pore volumes of all three treated coals were lower than that of the raw coal. The reduction was most pronounced for PG-coal, which showed a 14.2% decrease in cumulative volume (for pores up to 172 nm). Specifically, the volumes of 20–50 nm mesopores and 50–100 nm macropores in PG-coal decreased by 0.92 × 10^–3^and 0.68 × 10^–3^cm^3^/g, respectively, relative to the raw coal. The specific surface area of BHA-coal was 0.15 cm^2^/g higher than that of the raw coal, while the specific surface area of BHT-coal was the same as that of the raw coal. The specific surface area of PG-coal was 0.15 cm^2^/g lower than that of the raw coal.

**Table 4:** Pore Parameters of Different Coal Samples

	Pore volume (10^–3^ cm^3^/g)					
Coal sample	<20 nm	20–50 nm	50–100 nm	>100 nm	BET average pore diameter (nm)	BET specific surface area (cm^2^/g)
BHA-coal	2.12	3.32	3.03	2.61	24.90	2.12
BHT-coal	1.82	3.24	3.09	2.38	24.32	1.97
PG-coal	1.85	2.8	2.89	2.2	26.16	1.8
Raw-coal	1.84	3.72	3.57	2.08	28.34	1.97

In summary, while BHT had negligible impact on the coal’s physical structure and BHA even increased its specific surface area, PG effectively reduced both the specific surface area and cumulative pore volume of the coal. This result indicates that PG densifies the coal microstructure, thereby exerting a physical inhibition effect. This finding is consistent with thermal analysis results, where PG increased the characteristic temperature T_1_.

### 3.4 Effect of Inhibitor on Free Radical Reaction

The inhibitory effect of antioxidants on coal spontaneous combustion is widely attributed to their free radical-scavenging capability.^27,28^ EPR spectroscopy probes this by applying an external magnetic field to induce Zeeman splitting of energy levels in unpaired electrons. Resonance absorption occurs upon exposure to microwave radiation of matching energy, and the resulting signals are analyzed to determine the concentration and environment of free radicals. Figure[Fig fig10] shows that all coal samples exhibit similar spectral line shapes, each characterized by a single peak and trough without hyperfine structure. At all oxidation temperatures, the peak intensities of the inhibited samples are lower than that of raw coal, confirming that all three antioxidants effectively reduce the free radical concentration. The order of peak intensity is BHA-coal > BHT-coal > PG-coal, indicating that PG demonstrates the strongest radical-scavenging capacity, followed by BHT, which shows marginally better performance than BHA. This performance difference gradually diminishes as temperature rises, and at 200 °C, the EPR spectra of BHA-coal and BHT-coal converge, a finding consistent with the thermal analysis results. Furthermore, the peak positions of all inhibited samples shift to the right, suggesting that the antioxidants not only reduce the overall free radical content but also modify their chemical environment or reactivity.

**Figure 10:**
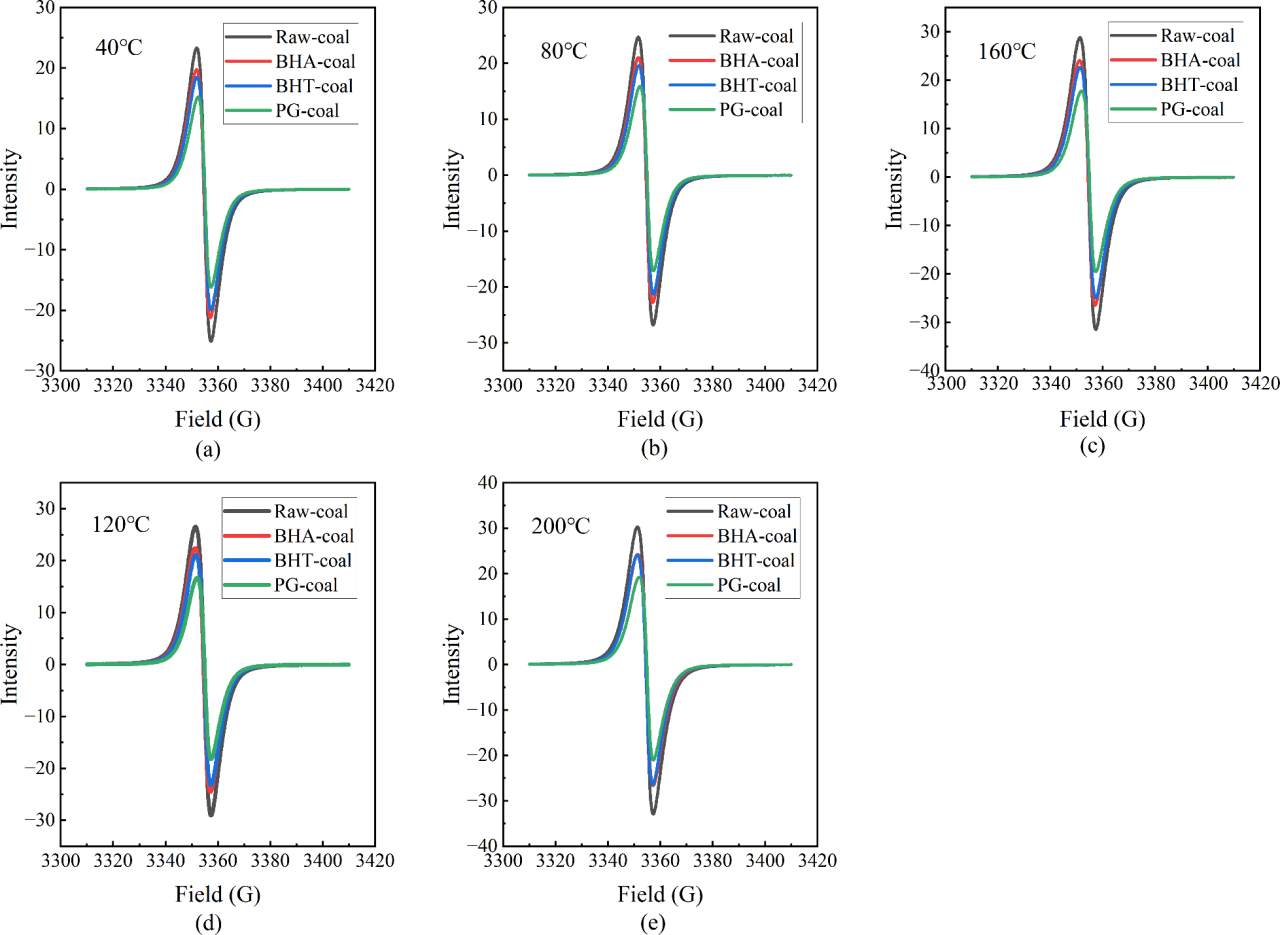
EPR spectra of coal samples at different oxidation temperatures: (a) 40 °C; (b) 80 °C; (c) 120 °C; (d) 160 °C; (e) 200 °C.

Figure 11 illustrates the variations in the g value, line width, and free radical concentration (Ng) of the coal samples with temperature. These characteristic parameters quantitatively reflect the evolution of free radicals. The key process in coal spontaneous combustion is the conversion of carbon-centered free radicals to oxygen-centered free radicals. Carbon-centered free radicals (e.g., ·CH_2_), generated initially, react rapidly with O_2_ to form peroxy radicals (ROO·). These ROO· radicals then initiate chain reactions, promoting further oxidation. The g-value reflects changes in the chemical environment during early coal oxidation, where a lower value indicates a suppressed conversion of carbon-centered free radicals to oxygen-centered free radicals. Figure 11(a) shows that the g value of the three inhibited coal samples are lower than those of raw coal, confirming that all three inhibitors effectively hinder this conversion. At 120 °C, the g value of PG-coal decreased significantly compared to the other samples, primarily because PG contains three phenolic hydroxyl groups. Consequently, compared to the monohydroxy antioxidants (BHA and BHT), PG exhibits a superior hydrogen-donating capacity and higher scavenging efficiency for hydroxyl and peroxy radicals.

**Figure 11:**
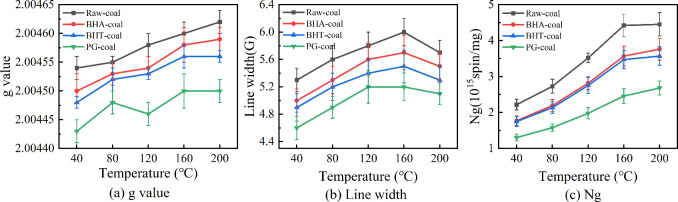
EPR characteristic parameters of coal samples at different temperatures: (a) g value; (b) line width; (c) Ng.

In EPR spectroscopy, line width, defined as the horizontal distance between the positive and negative peaks and reported in Gauss (G), reflects the strength of interactions between free radicals or between radicals and surrounding molecules. As shown in Figure 11(b), the line width of raw coal increases with temperature up to 160 °C, indicating that heating accelerates the generation and interaction of free radicals. Beyond this temperature, the line width decreases, a trend attributed to the excessive consumption of initially generated free radicals, which lowers their concentration and subsequently diminishes the intensity of radical reactions. Although the inhibited coal samples follow a similar trend, their line widths are consistently lower than that of raw coal across the temperature range. This demonstrates that the antioxidants effectively scavenge active free radicals within the coal matrix, thereby reducing the reaction intensity among radicals or between radicals and oxygen, and weakening the accelerating effect of temperature on coal oxidation.

Coal spontaneous combustion is fundamentally a chain reaction process, driven by the interaction of oxygen with active groups in the coal matrix. Within this process, free radicals serve as the key reactive centers, undergoing continuous generation and consumption. Consequently, monitoring the concentration of free radicals provides a direct measure of the combustion progression. As shown in Figure 11(c), all coal samples exhibit a similar trend in Ng. In the initial low-temperature oxidation stage (40–80 °C), coal physically adsorbs oxygen, generating a low concentration of alkyl radicals. As the temperature rises, the free radical concentration increases gradually. At elevated temperatures (80–160 °C), the breakage of side chains and branched structures in the coal molecules produces numerous oxygen-containing free radicals (e.g., ROO·, ·OH), leading to a rapid surge in concentration. In the high-temperature phase (160–200 °C), substantial gaseous products are generated while side chains and branched structures are rapidly consumed. This establishes a dynamic equilibrium between radical generation and consumption, causing the concentration growth to plateau. Critically, across the entire temperature range studied, the inhibited coal samples consistently exhibit lower free radical concentrations than the raw coal, demonstrating that all three inhibitors effectively mitigate the low-temperature oxidation process.

### 3.5 Effect of Inhibitor on Active Groups in Coal

During coal oxidation, active free radicals primarily originate from the cleavage of chemical bonds in functional groups. To further elucidate the inhibition mechanism of phenolic antioxidants, the evolutionary behavior of functional groups in the coal samples before and after inhibition was analyzed using in situ FTIR. The infrared spectrum of coal (Figure 12) can be divided into four primary regions: the hydroxyl region (3000–3700 cm^–1^), the aliphatic region (2800–3000 cm^–1^), the oxygen-containing functional group region (1000–1800 cm^–1^), and the aromatic structure region (400–900 cm^–1^).^29,30^ A comparison of the spectra from inhibited and raw coal samples revealed that while the overall spectral trends and absorption peak positions were largely consistent, discernible changes in peak shapes and areas were observed. This indicates that the inhibitors do not alter the types of functional groups present but rather influence the reaction pathways in which these groups participate.

**Figure 12:**
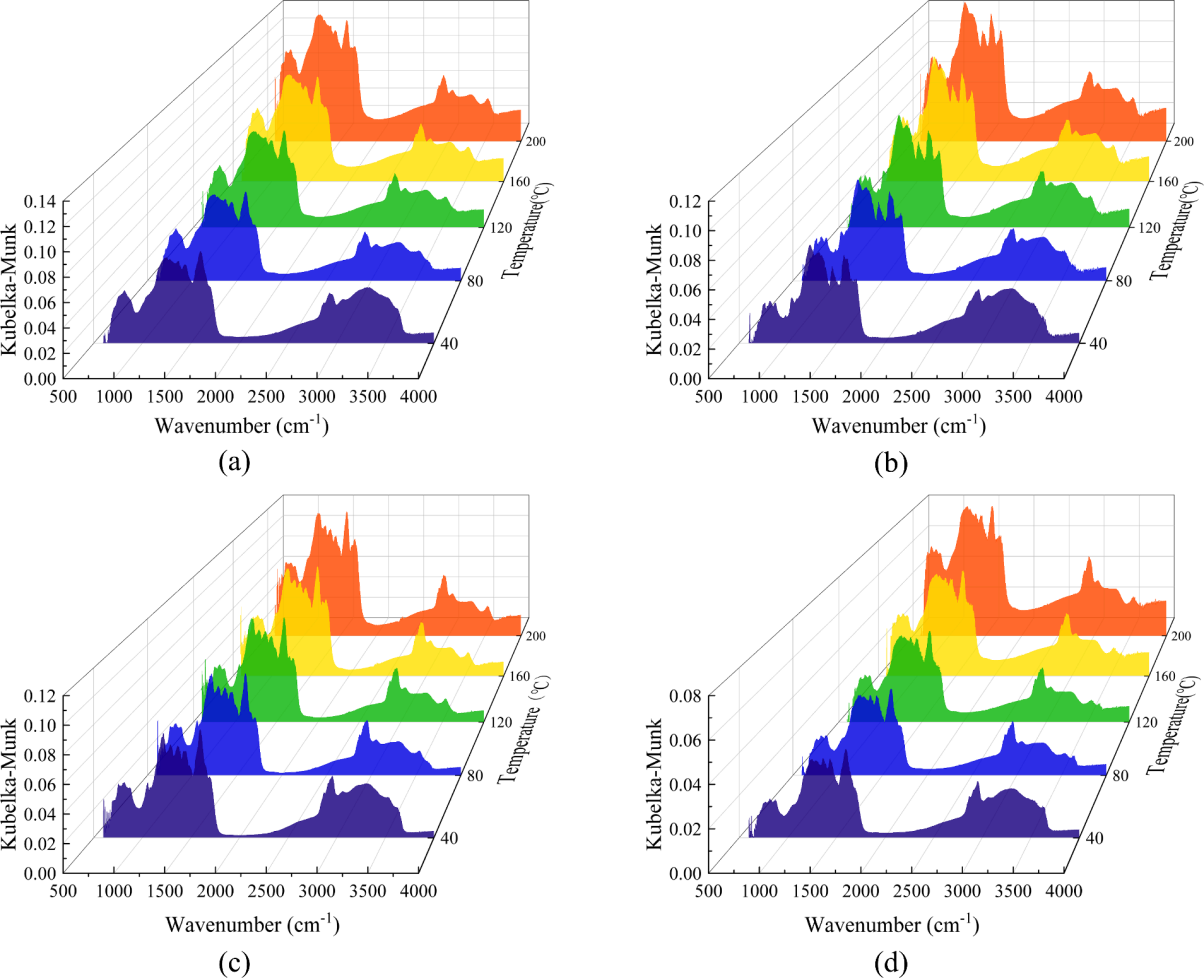
Infrared spectra of coal samples at different oxidation temperatures: (a) Raw-coal; (b) BHA-coal; (c) BHT-coal; (d) PG-coal.

During the low-temperature oxidation of coal, the aromatic core structures remain stable and do not participate in reactions. The characteristic stretching vibrations of functional groups associated with spontaneous combustion are primarily located in the 1000–1800 cm^–1^ and 2800–3700 cm^–1^ spectral regions. The observed peaks in these regions result from the overlap of multiple functional groups. To investigate how the inhibitors affect the reaction pathways of specific functional groups, peak fitting was performed in four key intervals: 1000–1550 cm^–1^, 1550–1800 cm^–1^, 2800–3000 cm^–1^, and 3000–3700 cm^–1^. The results of this deconvolution for raw coal at 40 °C are presented in Figure 13, which identifies the characteristic peak positions of different functional groups. Based on the fitting results across all temperatures, the peak areas for alkyl, alkoxy, carbonyl, and hydroxyl groups was quantified, as summarized in Figure 14.

**Figure 13:**
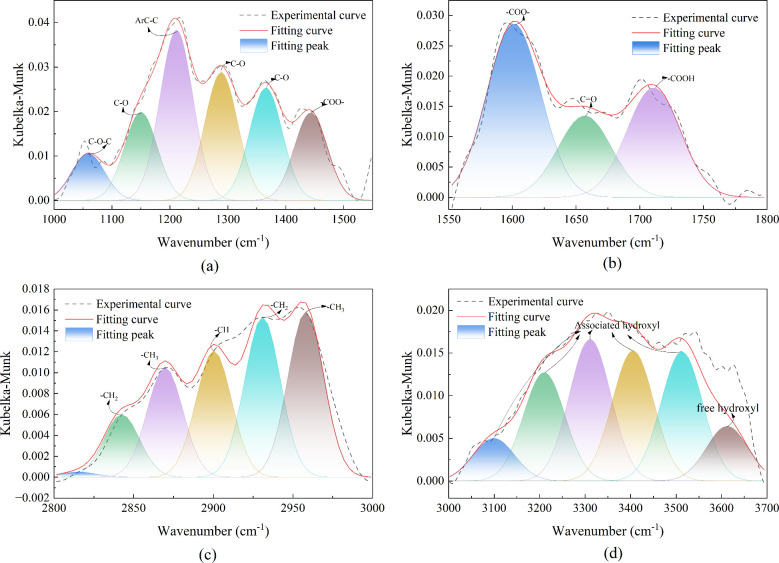
Fitting spectra of different functional groups: (a)­1000–1550 cm^–1^; (b) 1550–1800 cm^–1^; (c) 2800–3000 cm^–1^; (d) 3000–3700 cm^–1^.

**Figure 14:**
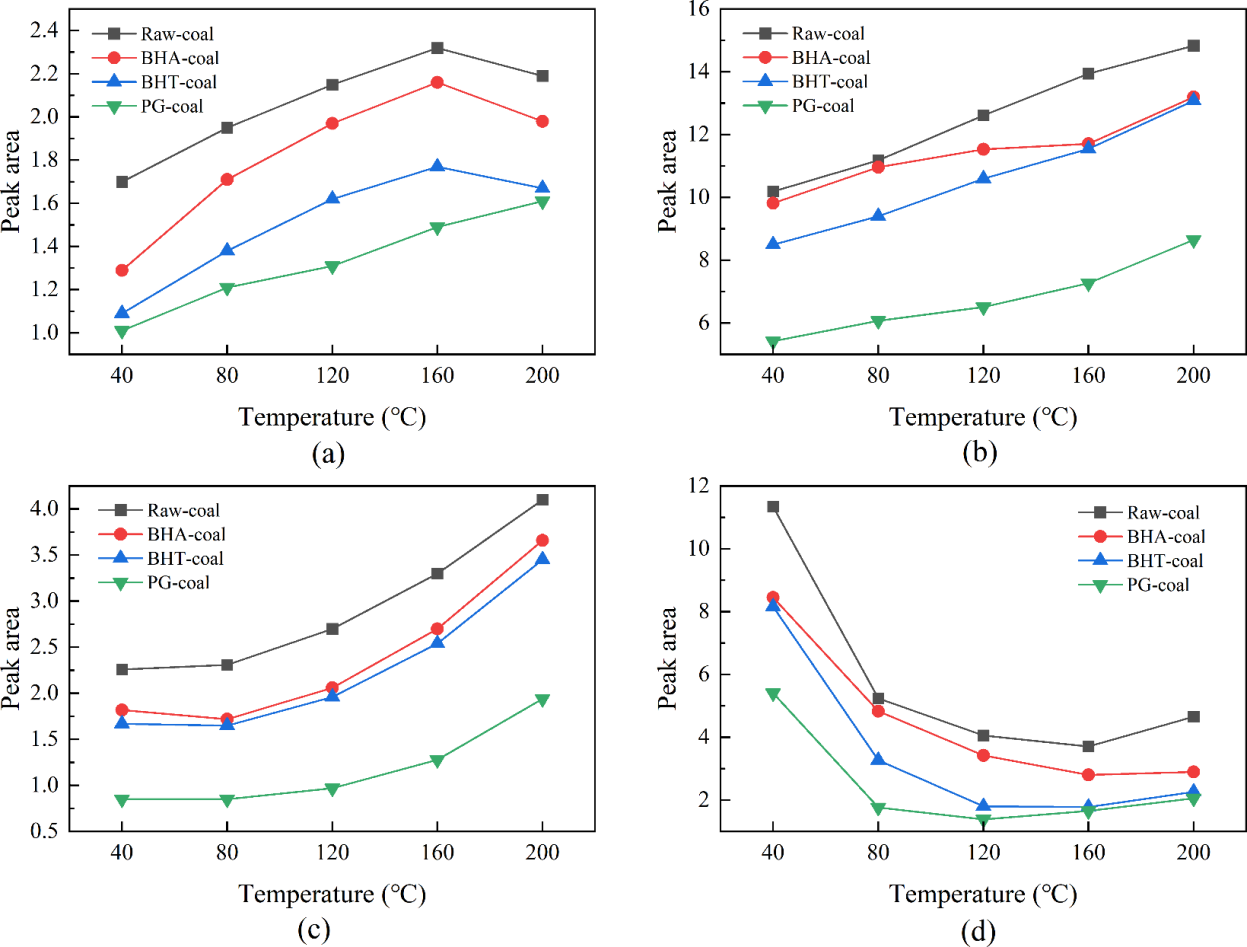
Characteristic peak area of functional groups in coal: (a) alkyl group; (b) alkoxy group; (c) carbonyl group; (d) hydroxyl group.

Figure 14(a) shows that the alkyl group content in raw coal first increases and then decreases with rising temperature. This trend can be explained by the molecular structure of coal. Bridge bonds containing heteroatoms, such as peroxy (−O-O−) and thioether (−C–S–C−) linkages, have low bond dissociation energies and preferentially cleave between 40 and 160 °C. This cleavage generates numerous alkyl fragments, leading to the observed increase in alkyl content. When the temperature reaches 160 °C, the oxidation reaction intensifies significantly. The increased activity of oxygen molecules enables them to overcome the reaction energy barrier, directly oxidizing the alkyl side chains of the coal molecules. Consequently, the dominant reaction pathway shifts from alkyl radical formation via bridge bond cleavage to the oxidative consumption of alkyl chains. As the consumption rate now surpasses the formation rate, the alkyl content decreases. Although the variation trend of alkyl content in the inhibited coal samples is similar to that of raw coal, their alkyl content is consistently lower at any given temperature. This indicates that phenolic antioxidants reduce the net yield of alkyl groups by inhibiting the radical chain reaction. Coal low-temperature oxidation is a typical free-radical chain process. Active sites in the coal structure first react with O_2_ to form peroxy radicals (−O-O·), which then attack the coal matrix, triggering subsequent reactions including bridge bond cleavage and alkyl oxidation. The phenolic hydroxyl groups in the antioxidants donate hydrogen atoms to highly active free radicals such as peroxy (−O–O·) and alkyl (−R·) radicals. This process generates stable phenoxy radicals, thereby terminating the chain reaction, inhibiting subsequent oxidation steps, and ultimately suppressing the accumulation of alkyl groups.

Below the coal pyrolysis threshold (40–200 °C), robust chemical bonds, including ether linkages and aromatic rings, remain largely intact. As shown in Figure 14(b), alkoxy group concentrations increase nearly linearly with rising temperature. Phenolic antioxidants inhibit this increase by scavenging active free radicals produced during coal oxidation, which disrupts the reaction pathways leading to alkoxy group formation. Consequently, the inhibited coal samples exhibit lower alkoxy group content than raw coal at the same temperatures.

During the low-temperature oxidation of coal, hydroxyl groups, unsaturated bonds, and aliphatic side chains react with oxygen, progressively transforming into carbonyl (C = O) structures, which constitute the primary source of the increasing carbonyl content. Figure 14(c) shows that the carbonyl content in raw coal evolves in two distinct stages. In the initial stage (before 120 °C), carbonyl formation occurs mainly through the dehydrogenation of alcohol hydroxyl groups. However, the low temperature results in a slow reaction rate, leading to only a minimal increase in content. Above 120 °C, the oxidation process intensifies, involving a greater number of functional groups and resulting in a rapid and substantial rise in carbonyl content. For instance, at these elevated temperatures, oxygen can attack the C–H bonds in long-chain alkyl groups, forming hydroperoxides (−CH­(OOH)−) that readily decompose into ketones (−CO−) or aldehydes (−CHO). While the temperature-dependent trend of carbonyl content is similar for the inhibited coal samples, their carbonyl content is consistently lower than that of raw coal at any given temperature. This suppression is primarily attributed to the phenolic antioxidants scavenging key free radicals (e.g., R·, ROO·) generated during coal oxidation. By intercepting these radicals, the inhibitors hinder the conversion of alkyl groups to hydroperoxides (ROOH), thus cutting off a major pathway for carbonyl group formation and ultimately leading to the observed reduction in carbonyl content.

Figure 14(d) shows that the hydroxyl content in raw coal exhibits three distinct stages of variation with temperature. In the first stage (40–80 °C), the content decreases rapidly due to the physical desorption of water molecules and the initial oxidation of weakly bound hydroxyl groups. In the second stage (80–160 °C), physically adsorbed water has been largely removed. The remaining hydroxyl groups are primarily chemically bound within the macromolecular structure (e.g., phenolic and alcoholic hydroxyls), requiring higher activation energy for reaction, which significantly slows their consumption rate. In the third stage (>160 °C), localized breakdown of the coal macromolecular structure occurs, leading to the generation of new hydroxyl groups that surpasses the consumption of original ones, resulting in a slight increase in overall content. The inhibited coal samples follow a similar trend, but their hydroxyl content is consistently lower than that in the raw coal at any given temperature. This confirms that temperature is the primary driver of the low-temperature oxidation process, with stage transitions governed by thermal energy thresholds. The role of phenolic antioxidants is to scavenge free radicals, thereby modulating the oxidation rate and consumption of hydroxyl groups, without altering the fundamental, temperature-driven reaction pathways.

### 3.6 Quantum Chemical Calculation

#### 3.6.1 Molecular Model Construction

The alkyl side chains in coal (e.g., −CH_3_, −CH_2_–, −CH = ) possess low bond dissociation energies, making them susceptible to cleavage under mechanical or thermal stress to form initial alkyl radicals (R·).^14^ These highly reactive R· radicals rapidly combine with oxygen to form peroxy radicals (ROO·). The ROO· radicals then attack C–H bonds in other alkyl groups, abstracting a hydrogen atom to generate hydroperoxides (ROOH) and regenerating an alkyl radical (R’·).^6^ This newly formed R’· radical propagates the chain reaction, leading to an exponential increase in the total radical population. Concurrently, the unstable ROOH intermediate decomposes slowly at low temperatures, yielding highly reactive alkoxy (RO·) and hydroxyl (·OH) radicals, which further accelerate the oxidation process.^31^

In essence, the low-temperature oxidation of coal is a radical chain reaction involving continuous generation, propagation, and branching. If uninterrupted, this autocatalytic process accelerates the oxidative degradation of coal, ultimately leading to spontaneous combustion. As established in previous studies,^32,33^ the O–H bond in phenolic antioxidants has a low dissociation energy due to resonance stabilization within the phenolic ring. This bond can readily dissociate, allowing the antioxidant to act as a hydrogen donor. By donating a hydrogen atom to active free radicals (e.g., R·, ROO·), the antioxidant neutralizes their unpaired electrons, forming stable products and effectively terminating the chain reaction.

To elucidate the inhibition mechanism of phenolic antioxidants, five key active radicals involved in the low-temperature oxidation of coal were selected for study: ·CH_3_, Ar–·CH_2_, Ar–CH_2_–O·, Ar–CH_2_–O–O·, and ·OH.^17,19,34^ The reactivity and thermodynamic parameters of these radicals with the antioxidants were then investigated computationally. Given the complexity of the full coal molecular structure and considering that the stable aromatic core does not significantly influence the reactivity of the side-chain functional groups, each radical was modeled by linking it to a benzene ring to represent the aromatic matrix of coal.^18^ The optimized structures of these molecular models are presented in Figure 15.

**Figure 15:**
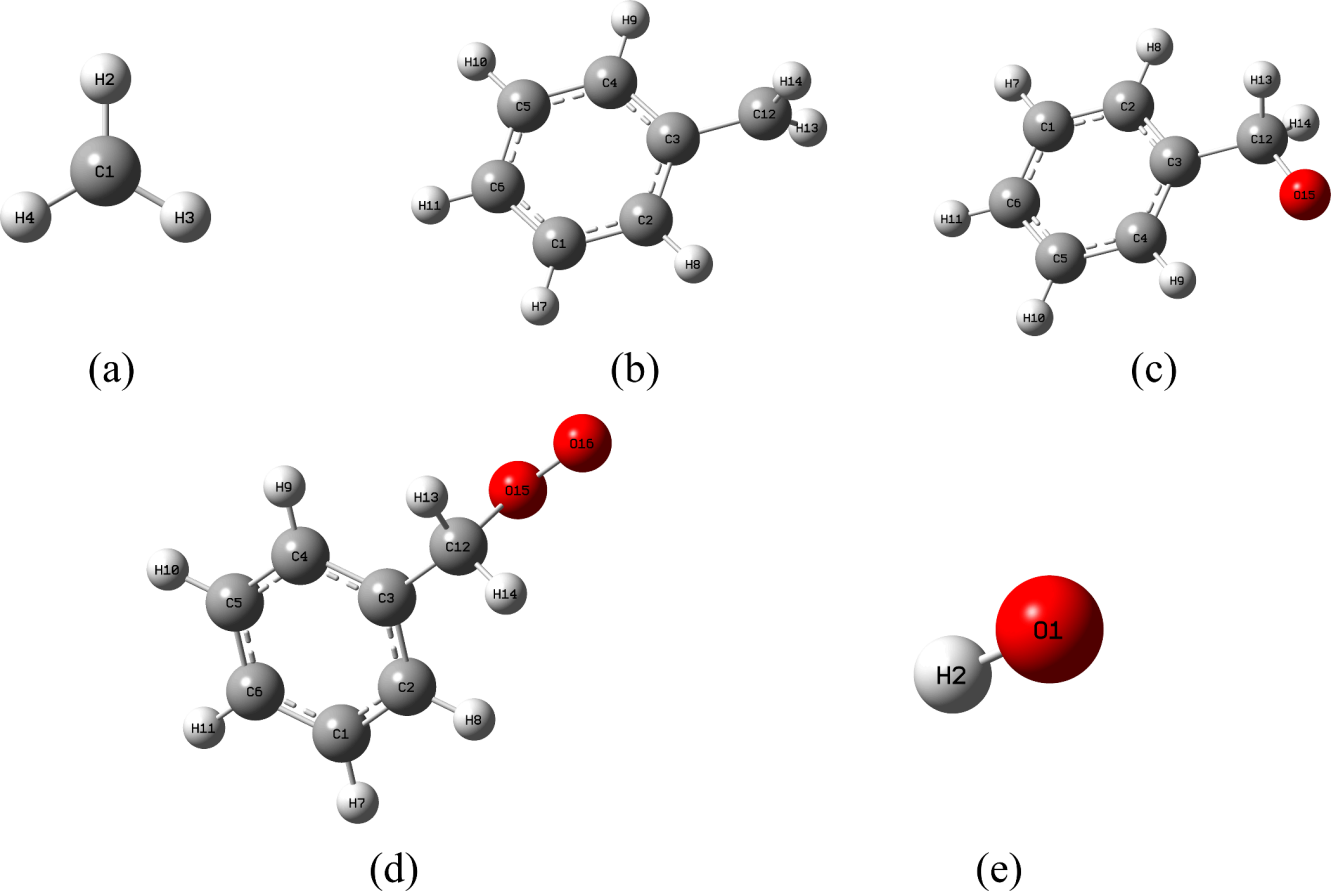
Molecular model of active groups: (a) ·CH_3_; (b) Ar–·CH_2_; (c) Ar–CH_2_–O·; (d) Ar–CH_2_–O–O·; (e) ·OH.

#### 3.6.2 Frontier Orbital Analysis

Frontier orbital theory, which focuses on the highest occupied and lowest unoccupied molecular orbitals (HOMO and LUMO) of reactants, provides a framework for understanding the mechanism and selectivity of chemical reactions. The reactivity between molecules is largely determined by the energy and symmetry properties of these frontier orbitals. For a reaction to proceed efficiently, two primary conditions must be satisfied. First, effective orbital overlap depends on symmetry matching between the frontier orbitals (e.g., the HOMO of one species and the LUMO of the other). Symmetry compatibility allows constructive overlap of wave functions, promoting bond formation, while incompatibility prevents effective interaction. Second, the energy gap between these orbitals must be sufficiently small to enable electron transfer; a difference of less than 6 eV typically favors reaction initiation.

In a free radical, the singly occupied molecular orbital (SOMO) contains the unpaired electron. For many organic radicals, the energy of the SOMO is comparable to that of a highest occupied molecular orbital (HOMO) in a closed-shell molecule and serves as the frontier orbital governing its reactivity. Phenolic antioxidants function as hydrogen atom donors. The reactivity of these molecules can be predicted by analyzing their frontier molecular orbitals; the sites of highest electron density in the HOMO typically indicate the locations most likely to participate in the reaction. Therefore, the interaction primarily depends on the overlap and energy difference between the antioxidant’s HOMO and the radical’s SOMO.

As shown in Figure 16, the highest electron density of the antioxidants’ HOMOs is predominantly located on the benzene rings. However, the extensive π-conjugation within the aromatic system leads to resonance stabilization, which delocalizes the electron density and results in lower reactivity compared to the hydroxyl side chains. In the SOMO of the methyl and methylene radicals, the electron density is primarily localized around the carbon atoms. In the absence of substituents, this distribution is symmetric. When linked to a benzene ring (as in Ar·CH_2_), conjugation occurs due to the parallel alignment of the carbon p-orbital with the ring’s π-system. This delocalizes the unpaired electron, shifting the density toward the aromatic ring. For oxygen-centered radicals, the SOMO electron density is concentrated on the oxygen atom. In the case of the hydroxyl radical (·OH), the electron density is almost entirely localized on oxygen, with a negligible contribution from the hydrogen atom. The HOMOs of the antioxidants and the SOMOs of the active radicals share the same irreducible representation. This symmetry match allows constructive overlap between the orbitals, increasing electron density in the interaction region and facilitating bond formation.

**Figure 16:**
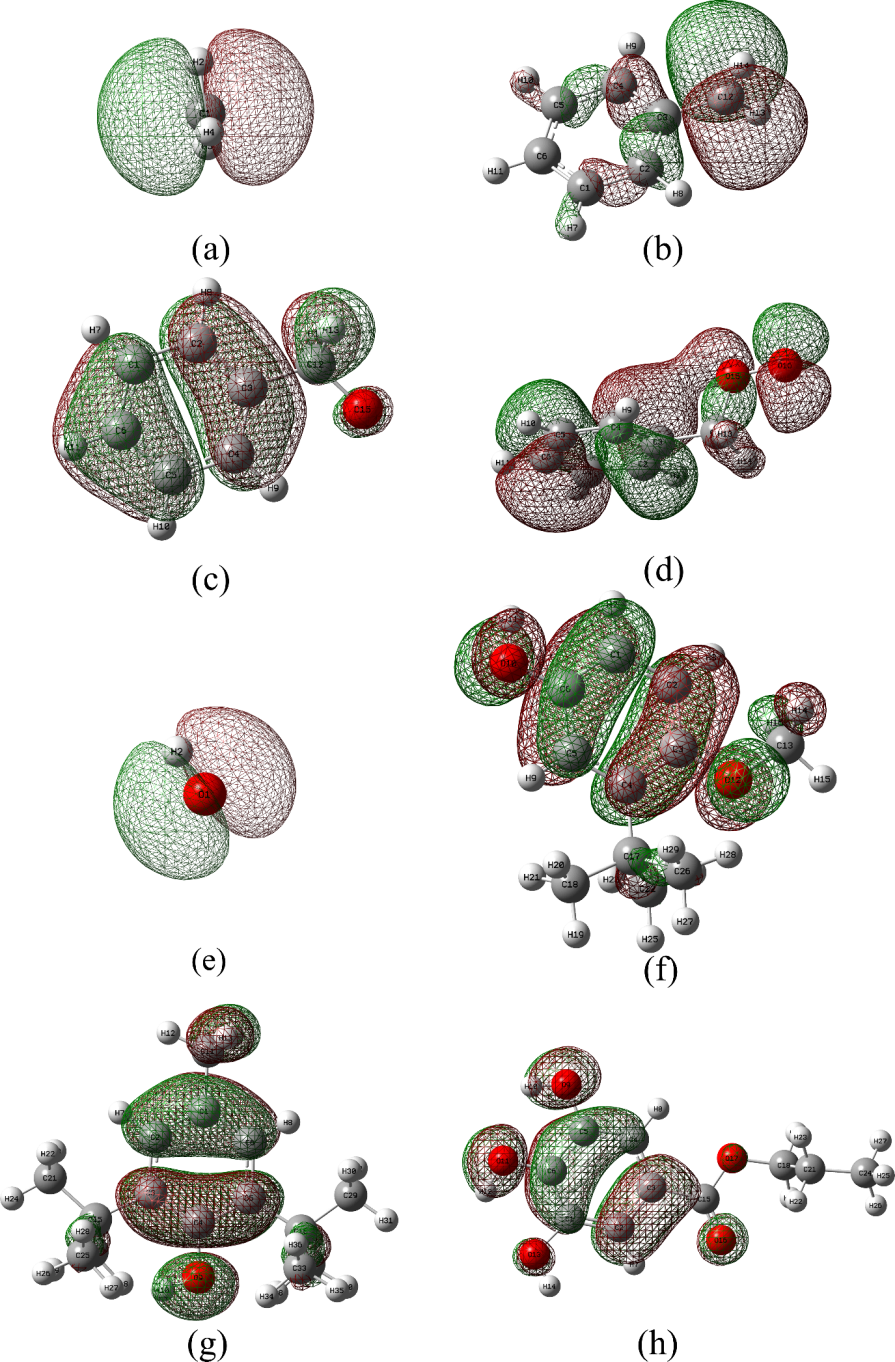
Electron cloud distribution of molecular frontier orbitals: (a) ·CH_3_; (b) Ar–·CH_2_; (c) Ar–CH_2_–O·; (d) Ar–CH_2_–O–O·; (e) ·OH; (f) BHA; (g) BHT; (h) PG.

As shown in Table 5, the energy gaps between the HOMOs of the three antioxidants and the SOMOs of the five active radicals are all below 6 eV. These small gaps facilitate electron transfer from the antioxidant (donor) to the radical (acceptor), indicating that the reactions are thermodynamically favorable. Therefore, all three phenolic antioxidants are predicted to be effective scavengers of these key radicals.

**Table 5:** Energy Gap between HOMO Orbital of Antioxidant and SOMO Orbital of the Active Group

	**Energy gap (eV)**		
**Active group**	**PG**	**BHA**	**BHT**
CH_3_·	0.00789	0.02967	0.02095
Ar–CH_2_·	–0.00059	0.02119	0.01247
Ar–CH_2_–O·	0.02823	0.05001	0.04129
Ar–CH_2_–OO·	0.04152	0.0633	0.05458
·OH	0.11004	0.13182	0.1231

Based on the above analysis, the reaction pathways between the three antioxidants and the five active radicals were predicted, as illustrated in Figure 17.

**Figure 17:**
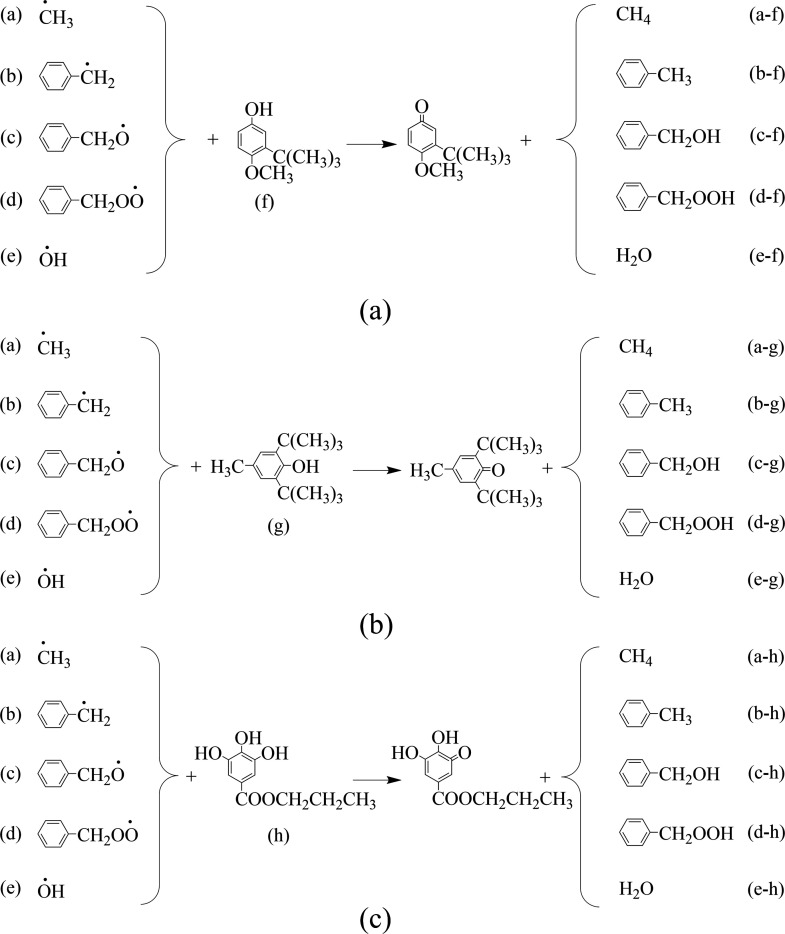
Reaction paths of antioxidants and active groups: (a) BHA and reactive group reaction pathway; (b) BHT and reactive group reaction pathway; (c) PG and reactive group reaction pathway.

#### 3.6.3 Analysis of Thermal Dynamic Parameters

To evaluate the reactivity between the antioxidants and active radicals, the enthalpy and Gibbs free energy for above reactions were calculated at room temperature using quantum chemical methods. The results are summarized in Table 6.

**Table 6:**
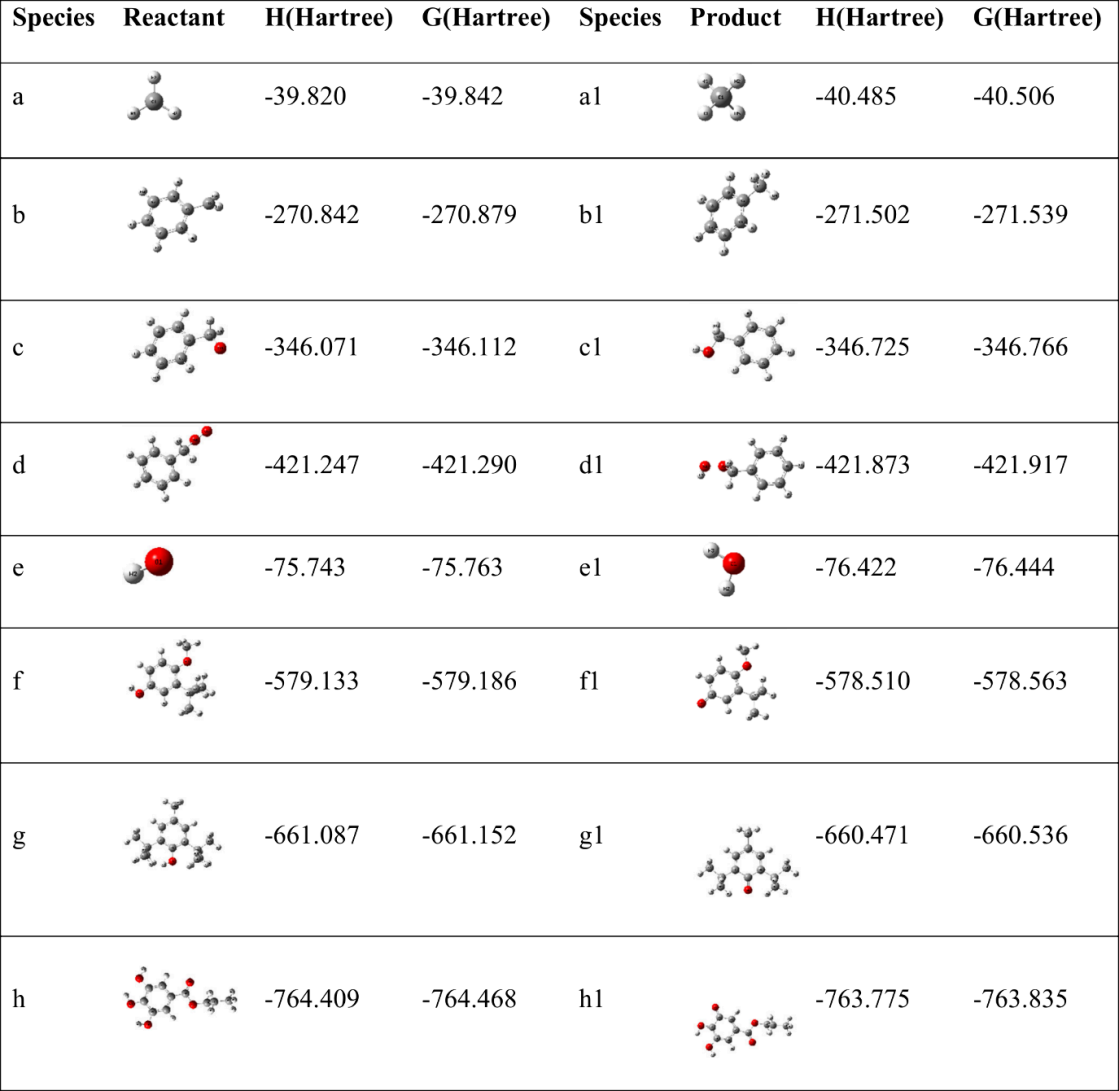
Enthalpy and Gibbs Free Energy of Reactants and Products after Structural Optimization

The enthalpy change (ΔH) represents the difference in total enthalpy between products and reactants, where a negative value signifies an exothermic reaction. In the context of radical scavenging, a more negative ΔH indicates greater energy release and higher stability of the resulting products, correlating with stronger antioxidant efficacy. The Gibbs free energy change (ΔG) describes the difference in Gibbs free energy between products and reactants and determines reaction spontaneity. A negative ΔG value indicates a spontaneous process, with more negative values reflecting a stronger thermodynamic driving force.

As shown in Table 7, the enthalpy and Gibbs free energy changes for reactions (d-h) are both greater than zero, indicating that the reaction between PG and the Ar–CH_2_–OO· radical is endothermic and nonspontaneous at room temperature. In contrast, the corresponding values for the other reactions are negative, confirming their spontaneity under ambient conditions. These results demonstrate that phenolic antioxidants can effectively scavenge the five key radicals generated during coal oxidation, thereby terminating the free radical chain reaction. The reactivity order of the phenolic antioxidants with the five key radicals was highly consistent: ·OH > CH_3_· > Ar–CH_2_· > Ar–CH_2_–O· > Ar–CH_2_–OO·. For reactions with the same radical, the relative reactivity among the three antioxidants followed the order BHT > BHA > PG, suggesting that BHT possesses the strongest inhibition potential according to the calculations, followed by BHA, with PG being the least effective. This theoretical prediction, however, contradicts the DSC experimental results. This discrepancy arises because the computational model considered only the most reactive hydroxyl group in PG. In practice, the two additional hydroxyl groups also contribute significantly to its overall scavenging capacity. Consequently, the actual inhibitory effectiveness of PG, as measured by DSC, exceeds the value predicted by the simplified computational model.

**Table 7:** Thermodynamic Calculation Results of Each Reaction

**Serial Number**	**ΔH** **(kJ/mol)**	**ΔG** **(kJ/mol)**
a-f	–110.615	–108.504
b-f	–94.764	–99.170
c-f	–80.644	–81.553
d-f	–8.237	–9.465
e-f	–148.445	–152.037
a-g	–129.282	–127.006
b-g	–113.431	–117.672
c-g	–99.311	–100.055
d-g	–26.903	–27.967
e-g	–167.111	–170.539
a-h	–83.709	–82.113
b-h	–67.858	–72.779
c-h	–53.738	–55.162
d-h	18.669	16.927
e-h	–121.539	–125.646

#### 3.6.4 Inhibition Mechanism

Phenolic antioxidants primarily inhibit coal spontaneous combustion through hydrogen atom transfer mechanism. In this process, the phenolic hydroxyl group donates a hydrogen atom, converting highly active free radicals into stable molecules (e.g., H_2_O, CH_4_, and alcohols) while the antioxidant itself forms a stable phenoxyl radical. Among the various radicals involved in coal-oxygen reactions, ·OH and CH_3_· are particularly reactive. They rapidly initiate chain reactions by abstracting hydrogen atoms from C–H bonds in the coal matrix, generating new radicals (Ar–CH_2_·) that play a critical role in accelerating the oxidation process. Phenolic antioxidants preferentially scavenge ·OH and CH_3_·, thereby blocking the initiation of new chain reactions by these highly active radicals and suppressing free radical proliferation at the source. A schematic illustration of this inhibition mechanism is provided in Figure 18.

**Figure 18:**
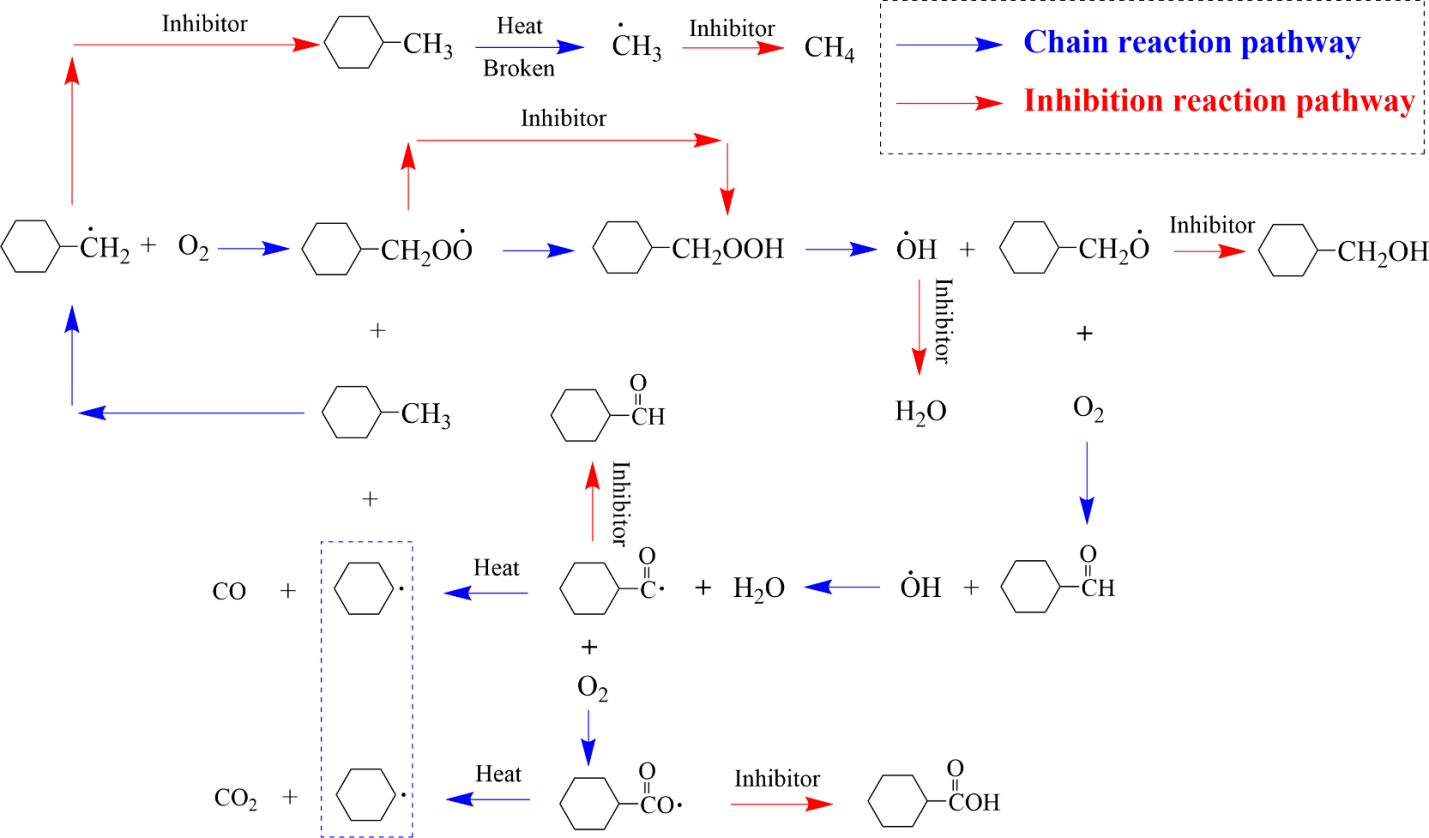
Inhibition mechanism of phenolic antioxidants.

BHT and BHA each possess a single phenolic hydroxyl group. Their high reactivity stems from steric hindrance around the phenolic site (in BHT) or the electron-donating effect of substituents (in BHA). In contrast, PG contains three phenolic hydroxyl groups. Although each hydroxyl in PG is individually less reactive than the single hydroxyl in BHT or BHA, one PG molecule can sequentially scavenge three free radicals by donating hydrogen atoms one after another. This multistep process affords PG a total radical scavenging capacity three times greater than that of monohydroxy phenols, enabling it to terminate chain reactions more comprehensively and exhibit superior overall inhibition. Furthermore, the polar hydroxyl and ester groups in the PG molecule facilitate strong adsorption onto the coal matrix via hydrogen bonding and dipole interactions. This adsorption allows PG to persist within coal pores, which reduces oxygen access to active sites and provides a physical inhibitory effect, particularly during the initial stages of coal oxidation.

## 4 Conclusions

Based on a comprehensive analysis of multiscale experimental data and quantum chemical calculations, the following conclusions are drawn regarding the inhibition effect and mechanism of phenolic antioxidants (PG, BHA, BHT) on coal spontaneous combustion:

(1) DSC and TPO experiments confirmed that the three antioxidants significantly increased the characteristic temperatures of coal samples and reduced CO release, demonstrating their macroscopic inhibitory effects. To address the limitations of single-method evaluations, this study established a comprehensive evaluation system based on the coal spontaneous combustion risk coefficient (C_r_) and destructive coefficient (C_d_). Under this evaluation, PG exhibited the best overall inhibition performance.
(2) SEM and LTNA results revealed that PG effectively reduced the specific surface area and cumulative pore volumes of coal, resulting in a more compact surface structure. Beyond chemical inhibition, PG also physically blocked oxygen diffusion into the coal, indicating a unique physicochemical synergistic inhibition mechanism.
(3) EPR and in situ FTIR analyses demonstrated that the phenolic antioxidants quenched active free radicals and suppressed the dynamic evolution of key functional groups (e.g., alkyl and carbonyl). These findings confirm that they acted as hydrogen donors, inhibiting coal spontaneous combustion by terminating radical chain reactions.
(4) Quantum chemical calculations confirmed that the reactions between phenolic antioxidants and key active radicals (e.g.,·OH) were spontaneous and exothermic. Integrating these findings with the macroscopic experimental results clarified that the number of hydroxyl groups and the steric hindrance effect were the key structural factors determining free radical scavenging efficiency. Thus, the structure–activity relationship was established, explaining the superior inhibition ability of PG at the molecular level by its trihydroxy structure.

In conclusion, this study confirmed that PG is an efficient, low-cost, and environmentally friendly inhibitor with strong potential to prevent coal spontaneous combustion. These findings provide a theoretical basis for its industrial application. However, it should be noted that these findings were based on experiments using a single type of coal (long-flame coal). Given the significant differences in physicochemical properties among coals of different ranks, the universality of phenolic antioxidants’ inhibitory effects requires further verification. Future work should systematically investigate samples across varying metamorphic degrees to clarify the general applicability of such inhibitors, thereby promoting their broader industrial adoption.
